# Phosphate (Bio)mineralization Remediation of ^90^Sr-Contaminated Groundwaters

**DOI:** 10.1021/acsestwater.3c00159

**Published:** 2023-08-31

**Authors:** Callum Robinson, Samuel Shaw, Jonathan R. Lloyd, James Graham, Katherine Morris

**Affiliations:** † Research Centre for Radwaste Disposal and Williamson Research Centre for Molecular Environmental Science, Department of Earth and Environmental Sciences, The University of Manchester, Manchester M13 9PL, U.K.; ‡ National Nuclear Laboratory, Sellafield, Cumbria CA20 1PG, U.K.

**Keywords:** bioremediation, in situ, mineralization, groundwater, radiostrontium

## Abstract

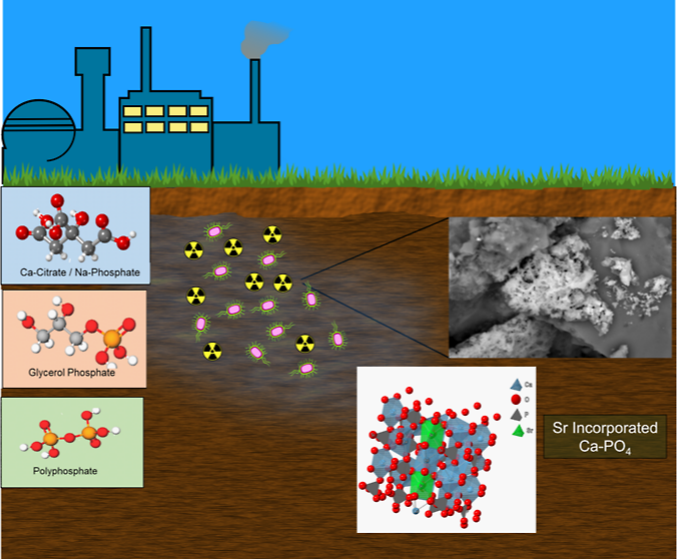

Historical operations at nuclear mega-facilities such as Hanford, USA, and Sellafield, UK have led to a legacy of radioactivity-contaminated land. Calcium phosphate phases (e.g., hydroxyapatite) can adsorb and/or incorporate radionuclides, including ^90^Sr. Past work has shown that aqueous injection of Ca-phosphate-generating solutions into the contaminated ground on both laboratory and field scales can reduce the amount of aqueous ^90^Sr in the systems. Here, two microbially mediated phosphate amendment techniques which precipitated Ca-phosphate, (i) Ca-citrate/Na-phosphate and (ii) glycerol phosphate, were tested in batch experiments alongside an abiotic treatment ((iii) polyphosphate), using stable Sr and site relevant groundwaters and sediments. All three amendments led to enhanced Sr removal from the solution compared to the sediment-only control. The Ca-citrate/Na-phosphate treatment removed 97%, glycerol phosphate 60%, and polyphosphate 55% of the initial Sr. At experimental end points, scanning electron microscopy showed that Sr-containing, Ca-phosphate phases were deposited on sediment grains, and XAS analyses of the sediments amended with Ca-citrate/Na-phosphate and glycerol phosphate confirmed Sr incorporation into Ca-phosphates occurred. Overall, Ca-phosphate-generating treatments have the potential to be applied in a range of nuclear sites and are a key option within the toolkit for ^90^Sr groundwater remediation.

## Abstract 2

Aqueous strontium is sequestered in microcosm remediation experiments amended with phosphate through incorporation into low-solubility calcium phosphate mineral phases.

## Introduction

Legacy operations at global nuclear facilities, including Sellafield, UK, Hanford, USA, and Mayak, Russia, have resulted in a significant legacy of radioactively contaminated land and groundwater. Key radionuclide contaminants at these sites include ^238^U, ^99^Tc, ^137^Cs, and ^90^Sr, which are often present as co-contaminants in groundwater plumes. In particular, ^90^Sr is significant in groundwaters, including at the Sellafield nuclear facility.^1−4^ Migration of radionuclides in the subsurface may present an environmental hazard and in situ remediation techniques, which can non-invasively treat the subsurface at these often densely packed, inaccessible, and complex sites, offer a promising approach to site remediation.^5^ In situ phosphate mineralization techniques, which are based on the enhanced sequestration of metal ions by Ca-phosphate phases,^5−8^ have been applied to ^90^Sr remediation in groundwaters^4,9^ and also show promise for remediation of U.^8,10−12^ In the environment, ^90^Sr exists as the Sr^2+^ cation and has similar geochemical behavior to Ca^2+^ in the aqueous phase. The principal mechanism of Sr^2+^ retention in the natural environment at circumneutral pH is adsorption via the formation of outer sphere sorption complexes to, e.g., clays and hydrous ferric oxides.^13−17^ This implies ^90^Sr^2+^ may be susceptible to (re)mobilization on alteration of subsurface pH and ionic strength.^17^ In addition, Sr^2+^ can incorporate into Ca-bearing minerals such as calcite (CaCO_3_) or Ca-phosphate phases, including, e.g., hydroxyapatite (Ca_5_(PO_4_)_3_(OH)) during their formation.^8,18,19^ For example, Sr^2+^-incorporated calcite formation can be promoted by hydrolyzing urea with ureolytic bacteria and producing ammonium and carbonate, resulting in an increased pH and the (bio)precipitation of calcite with resultant Sr co-precipitation and incorporation.^18,20^ Additionally, Ca-phosphate biomineralization has been used successfully to sequester ^90^Sr from groundwaters by promoting in situ formation of calcium phosphate minerals such as hydroxyapatite.^4^ Notably, Sr^2+^ incorporation into Ca-phosphates makes the Sr^2+^ less susceptible to ion-exchange reactions and provides a more stable end point than simple outer sphere sorption alone due to the typically low solubility of Ca-phosphates including hydroxyapatite.^21,22^ Ca-phosphate remediation approaches have focused on the slow (microbially mediated) release of either calcium or phosphate into the subsurface groundwater zone to be treated to induce Ca-phosphate formation while preventing instantaneous precipitation and injection well clogging.^23^ Indeed, chemical and biological methods which promote Ca-phosphate formation have been deployed at nuclear-licensed sites to achieve ^90^Sr remediation.^4,24^ At the Hanford nuclear facility, USA, both biotic and abiotic phosphate mineralization approaches were used to successfully remediate ^90^Sr and U contaminated groundwater through formation of both ^90^Sr-substituted Ca-phosphates and poorly soluble U(VI) phosphate phases.^4,24^ During remediation of the ^90^Sr groundwater plume within the 100N area of the Hanford site, injection of solutions containing Ca-citrate and Na-phosphate were used to promote Ca-phosphate biomineralization while minimizing borehole clogging.^25,26^ Here, biotic breakdown of citrate-chelated calcium under oxic conditions, facilitated the slow release of aqueous Ca^2+^ into solution. Subsequently, the Ca^2+^_(aq)_ reacted with inorganic phosphate, which was co-injected as soluble Na-phosphate, to precipitate Ca-phosphate (hydroxyapatite-like) phases. Degradation of citrate is driven by a variety of microbial processes during which citrate is expected to undergo oxidation to carbon dioxide or partial oxidation to products including acetate, formic acid, and formaldehyde.^27^ Initially, studies highlight that the resultant biogenic Ca-phosphate phases are amorphous and initial ^90^Sr removal is dominated by adsorption to these poorly ordered precipitates. In the long term, over weeks to months, the crystallinity of the Ca-phosphates increases, with initially adsorbed Sr^2+^ becoming partially incorporated into the Ca-phosphate lattice through isomorphous exchange with Ca^2+^ to form more ordered and thermodynamically stable Sr^2+^ substituted hydroxyapatite with time.^4^ Furthermore, due to the presence of two distinct Ca^2+^ sites, hydroxyapatite is able to flexibly incorporate different radionuclides, including ^90^Sr, U, and Th, suggesting that it may be able to co-treat several contaminants.^7,28,29^

Abiotic mineralization approaches have also been investigated at the Hanford nuclear site, using polyphosphate amendment solutions to remediate U-contaminated land.^24^ Here, a mix of readily available orthophosphate (PO_4_^3–^) and less available pyrophosphate (P_2_O_7_^4–^) was injected into the subsurface to promote Ca-phosphate mineral precipitation.^24,30,31^ When injected, the reaction between groundwater Ca^2+^_(aq)_ and orthophosphates formed low solubility U-bearing calcium phosphate phases. Ongoing precipitation of Ca-phosphates was then thought to be sustained by slow hydrolysis of the pyrophosphate, liberating additional orthophosphate which further reacted with aqueous Ca^2+^ enabling Ca-phosphate precipitation within a wider area of the subsurface.^32^

More recently, glycerol phosphate has been explored as both a slow-release, readily biodegradable phosphate source, and electron donor for indigenous microorganisms to facilitate the formation of Ca-phosphate biominerals and (bio)reducing conditions.^8,10,11,33^ Here, phosphatase initially cleaved the glycerol phosphate, liberating inorganic phosphate into solution which then reacted with Ca^2+^ in solution to precipitate poorly soluble Ca-phosphate phases. Sr was then sorbed and incorporated into the Ca-phosphate phases.^8,34^ Previous work has also investigated the removal of uranium in glycerol phosphate-stimulated microcosms.^10^ Here, the addition of glycerol phosphate caused U(VI) removal from solution through reduction of U(VI) to U(IV) followed by precipitation of recalcitrant, U(IV)-phosphate phases.^10^

Although past work has demonstrated the potential use of these remediation approaches, their applicability under a wide range of biogeochemical conditions has yet to be fully explored. In addition, consideration of tailoring the amendment techniques to site-specific conditions, for example, via addition of Ca^2+^ to the remediation solutions, and the chemical speciation of sequestered Sr^2+^ within the different treatments is also relatively poorly constrained.

We aimed to explore the applicability of these approaches (Ca-citrate/Na-phosphate,^4^ glycerol phosphate,^8^ and polyphosphates^35^) to sequester Sr^2+^ under oxic conditions representative of the Sellafield, UK, nuclear-licensed site. Our objectives were to set up microcosm experiments using representative sediments and synthetic groundwater and investigate the removal mechanism of Sr during phosphate mineralization. We used a range of analytical techniques to determine the speciation and fate of Sr during the different treatments. Overall, we show that removal of Sr^2+^_(aq)_ from the solution was enhanced via adsorption to precipitated, poorly ordered Ca-phosphate phases in all three techniques, with some evidence for partial Sr^2+^ incorporation into the newly formed minerals and the Ca-citrate/Na-phosphate amendment removing most Sr^2+^ from solution.

## Materials and Methods

### Sediment Collection and Characterization

Sediments representative of the subsurface at Sellafield were collected in December 2019. The first was a relatively clay-rich glacial till (Calder River; 54°26′28.8″N 3°28′10.8″W^36^), and the second was a relatively clay-poor outwash sand (Peel Place Quarry; 54°23′49.2″N 3°25′59.9″W^37^). On sampling, sediments were immediately sealed in sterile HDPE bags and stored in the dark at 10° ± 1 °C prior to use in microcosms. The bulk mineralogical composition was determined using X-ray diffraction (XRD) (Bruker D8 Advance). Elemental analysis of Peel Place Quarry (PPQ) sediment was achieved using X-ray fluorescence (XRF) (PANalytical Axios). BET surface area measurement was performed using Micromeritics Gemini V (model 2365).

### Sr Biomineralization Microcosms

Experiments were set up with a 1:10 sediment-to-synthetic groundwater ratio in sterile 500 mL conical flasks. The synthetic groundwater recipe was informed by authentic site groundwater data (Supporting Information Section S1). Here, the distribution of the major ions within the groundwater was calculated and an average composition of the Sellafield groundwater was computed (Supporting Information Section S1). This enabled synthetic groundwater to be synthesized from dissolution of the relevant salts (see Supporting Information Section S1 and Table S2) comprising in mM: 0.21 Mg^2+^_(aq)_, 0.69 Ca^2+^_(aq)_, 1.5 Na^+^_(aq)_, 0.07 K^+^_(aq)_, 0.28 SO_4_^2–^_(aq)_, 0.32 NO_3_^–^_(aq)_, 1.51 Cl^–^_(aq)_, and 0.98 HCO_3_^–^_(aq)_. In addition, stable Sr^2+^ was added to the groundwater at 1 mM (88 ppm), which was undersaturated in synthetic groundwater (confirmed by geochemical modeling PHREEQC version 3, see below^38^) at pH 6.5, the initial pH of the groundwater (Section S1). The experimental Sr concentration of 1 mM was selected to allow direct spectroscopic and solid phase chemical analysis of the Sr solids in the microcosms while retaining some relevance to the expected subsurface concentration of both stable and (ultra-trace) ^90^Sr at Sellafield. Stable Sr^2+^ in regional groundwater is reported at 0.12 ppm (1.4 μM),^39^ with representative upper range reported ^90^Sr concentrations of ∼59,000 Bq/L (∼0.1 nM),^2^ suggesting ^90^Sr will have a negligible contribution to the total Sr concentration in groundwaters. The concentration of 1 mM/88 ppm Sr^2+^ was therefore chosen to target a final solid phase Sr concentration of several hundred ppm, an order of magnitude above background concentrations in sediments (64 ppm; Table S3). The synthetic groundwater was then sterilized and re-adjusted to pH 6.5 using HCl.

Batch microcosm experiments exploring the removal of soluble Sr^2+^ with the following amendments: (i) Ca-citrate/Na-phosphate, (ii) glycerol phosphate, and (iii) polyphosphate using both PPQ and Calder River (CR) sediments were undertaken.

For the Ca-citrate/Na-phosphate system, CaCl_2_·2H_2_O and Na_3_C_6_H_8_O_7_·2H_2_O were dissolved in deionized water to give a concentrated stock solution containing 50 mM Ca^2+^ and 125 mM Citrate. We also prepared a concentrated stock of 100 mM phosphate solution created from dissolution of Na_2_HPO_4_·H_2_O. These concentrated stocks were then spiked to microcosms to achieve a range of different aqueous Ca^2+^, citrate, and phosphate treatment concentrations as informed by past work.^26^ These were, for PPQ sediments, 1 mM (1.69 mM total) Ca^2+^, 2.5 mM citrate, 5 mM (5.69 mM total) Ca^2+^, 12.5 mM citrate, and 10 mM phosphate, and for CR sediment, 2 mM (2.69 mM total) Ca^2+^ and 5 mM citrate with 10 mM phosphate. Here, the stability of the Ca-citrate / Na-phosphate amendments in the absence of sediment microbial populations has been reported as up to 7 days.^25^ For the glycerol phosphate amended system, 10 mM filter sterilized glycerol phosphate was spiked into the PPQ and CR microcosms. The polyphosphate experiments were conducted with 10 mM polyphosphate consisting of 90% orthophosphate (mixture of KH_2_PO_4_, K_2_HPO_4_, NaH_2_PO_4_, and Na_2_HPO_4_) and 10% pyrophosphate (Na_4_P_2_O_4_), which was added to the PPQ and CR microcosms.^24^

Once spiked with the appropriate amendments, flasks (500 mL) were capped with porous foam bungs to maintain an oxic headspace in equilibration with laboratory air and stored under dark conditions at room temperature for 31 days, with gentle swirling at least twice per week to an avoid excessive abrasion consistent with past studies.^8,40^ All experiments were run in triplicate and sediment-only controls with 1 mM (88 ppm) Sr^2+^ were also prepared.

### Geochemical Analysis

Periodically, microcosms were sampled using an aseptic technique, and the resultant slurry was centrifuged (8000 rpm, 10 min) to separate the solution and solids. Aqueous samples were analyzed for pH (Jenway 3520 pH meter equipped with a Fisherbrand FB68801 electrode), and a subaliquot was prepared for inductively coupled plasma atomic emission spectroscopy analysis of Sr and Ca by dilution into 2% HNO_3_ (Perkin Elmer Optima 5300). Samples for ion chromatography were stored in a fridge at 4 °C prior to analysis for citrate, glycerol phosphate, and acetate (Dionex ICS 5000). The inorganic phosphate concentration in the solution was determined at the point of sampling via spectrophotometric assay.^41^ To further explore likely strontium speciation in these experiments, thermodynamic modeling using PHREEQC version 3^38^ using the ThermoChimie (V10a) database^42^ was conducted using selected aqueous data from the experimental systems (Section S3).

### 16S Ribosomal (rRNA) Gene Analysis

DNA was extracted from sediment endpoints using a DNeasy PowerSoil Pro Kit (Qiagen, Manchester, U.K) following the method detailed by Foster et al., 2020.^43^ Sequencing of PCR amplicons of 16S rRNA genes was conducted with the Illumina MiSeq platform (Illumina, San Diego, CA, USA), targeting the V4 hypervariable region (forward primer, 515F, 5′-GTGYCAGCMGCCGCGGTAA-3′; reverse primer, 806R, 5′-GGACTACHVGGGTWTCTAAT-3′) for 2 × 250-bp paired-end sequencing (Illumina).^44,45^ PCR amplification was performed using a Roche FastStart High Fidelity PCR System (Roche Diagnostics Ltd, Burgess Hill, UK) in 50 μL reactions as outlined by Foster et al., 2020,^43^ with initial denaturation at 95 °C for 2 min, followed by 36 cycles of 95 °C for 30 s, 55 °C for 30 s, 72 °C for 1 min, and a final extension step of 5 min at 72 °C. PCR products were purified and normalized to approximately 20 ng each using a SequalPrep Normalization Kit (Fisher Scientific, Loughborough, UK). PCR amplicons from all samples were pooled in equimolar ratios. The run was performed using a 4.5 pM sample library spiked with 4.5 pM PhiX to a final concentration of 12%.^46^ After amplification and sequencing, the raw sequences were divided into samples by barcodes (up to one mismatch was permitted), using a sequencing pipeline. Quality control and trimming were performed using Cutadapt,^47^ FastQC,^48^ and Sickle.^49^ MiSeq error correction was performed using SPADes.^50^ Forward and reverse reads were incorporated into full-length sequences with Pandaseq,^51^ and chimeras were removed using ChimeraSlayer following the method of Foster et al., 2020.^43,52^ OTU’s were generated with UPARSE,^53^ and classified by Usearch^54^ at the 97% similarity level, and singletons were removed. Rarefaction analysis was conducted using the original detected OTUs in Qiime.^55^ The taxonomic assignment was performed by the RDP naïve Bayesian classifier version 2.2,^56^ used in combination with the Silva SSU 132 ribosomal RNA gene database.^57^ The OTU tables were rarefied to the sample containing the lowest number of sequences, all samples having less than 5000 sequences were removed from analyses prior to the rarefaction step. The step size used was 2000 and 10 iterations were performed at each step.

### X-ray Absorption Spectroscopy

To directly determine the speciation of Sr in sediments, X-ray absorption spectroscopy (XAS) was conducted on selected samples after 31 days of incubation. Here, PPQ sediments amended with (i) Ca-citrate/Na-phosphate (1 mM Ca^2+^, 2.5 mM citrate, and 10 mM phosphate), (ii) glycerol phosphate, and a sorption control with groundwater and Sr were prepared for XAS by mounting a moist sediment pellet into a cryovial and storing at −80 °C prior to analysis. Sr K-edge XAS analyses were performed using a liquid nitrogen-cooled cryostat at beamline B18, Diamond Light Source Harwell, UK. Spectra were collected in fluorescence mode using a solid-state detector. We also analyzed a Sr-incorporated hydroxyapatite standard prepared following the methods of Afshar et al., 2003 and Catros et al., 2010.^58,59^ The standard was analyzed at the Institute for Nuclear Waste Disposal (INE) beamline, Karlsruhe Institute of Technology, Germany. Data fitting was performed using ATHENA and ARTEMIS.^60^ Statistical evaluation of EXAFS refinement fitting (F testing) was conducted following the method of Downward et al., 2007.^61^

### Scanning Electron Microscopy

For SEM analyses, a small aliquot of sediment slurry was dried and mounted on SEM stubs. SEM scans with energy dispersive X-ray spectra (EDS) were collected using a FEI Quanta 650 FEG ESEM in low vacuum mode.

## Results and Discussion

### Sediment Characterization

XRD of the PPQ and CR sediments confirmed that they were dominated by quartz (SiO_2_), with some feldspars [albite (NaAlSi_3_O_8_)] and mica [muscovite (KAl_2_(AlSi_3_O_10_)(F,OH)_2_], and minor amounts of pyrite (FeS_2_) and clinochlore [Mg_5_Al(AlSi_3_O_10_)(OH)_8_] (see Section S2). XRF analysis confirmed that the major elemental composition of PPQ was SiO_2_ (90.4%), Al_2_O_3_ (4.30%), K_2_O (1.90%), Fe_2_O_3_ (1.40%), MgO (0.70%), and Na_2_O (0.60%) (Table S3). CR sediment was previously characterized as a uniformly graded sandy loam (53% sand, 42% silt, and 5% clay).^36^ Surface area measurements by BET were 1.76 ± 0.01 and 3.85 ± 0.02 m^2^ g^–1^ for PPQ and CR sediments, respectively. These analyses reflect that PPQ was a relatively clay-poor outwash sand,^37^ and CR was a clay- and silt-rich fluvioglacial deposit,^62^ with both of these lithologies representative of the heterogeneous Sellafield subsurface geology.^37^

### Microcosm Aqueous Biogeochemistry

#### Sorption Controls

**Figure 1:**
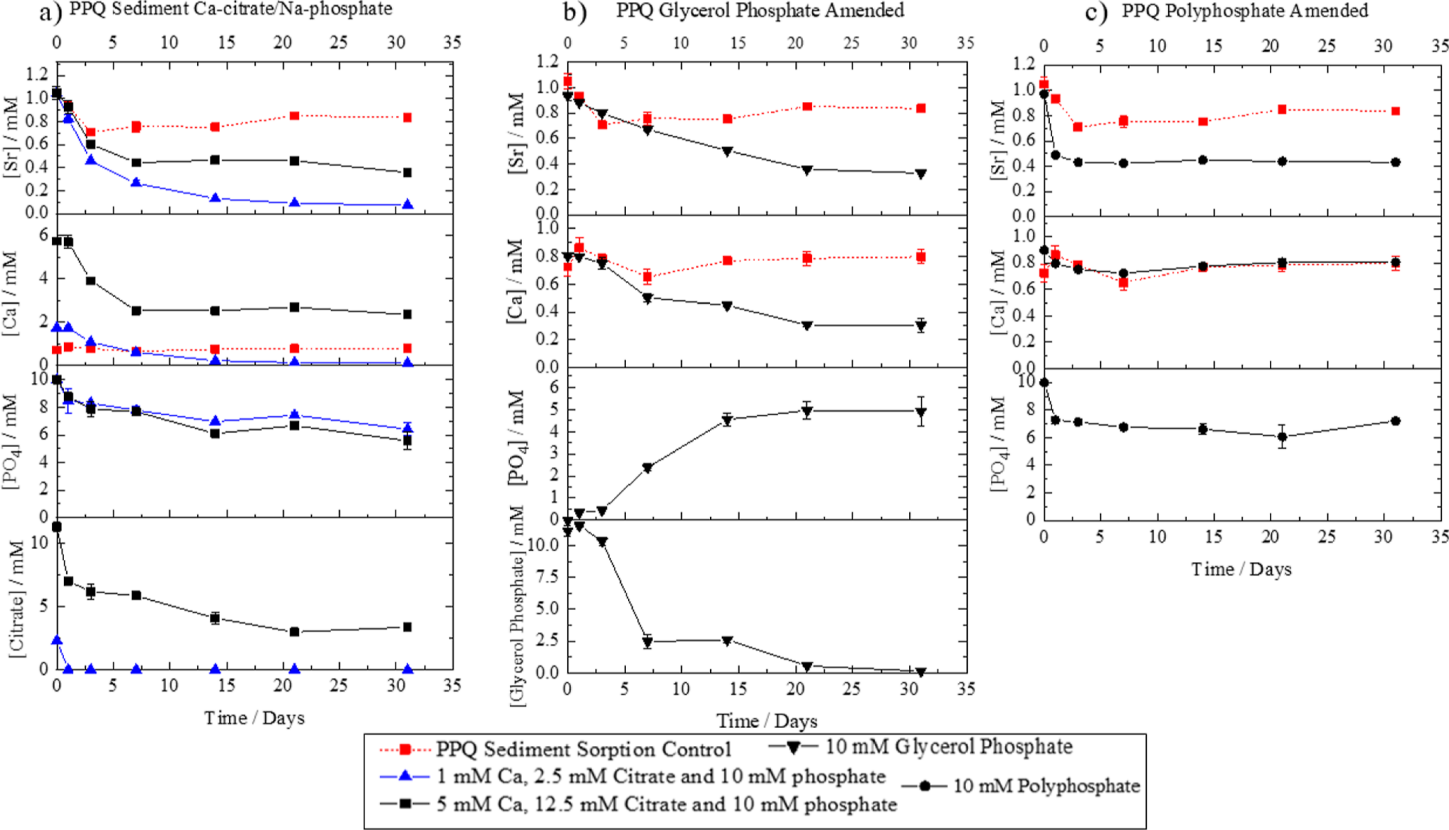
Aqueous geochemical data for the PPQ sediment experiments amended with (a) Ca-citrate/Na-phosphate, (b) glycerol phosphate, and (c) polyphosphate and co-plotted with the PPQ sediment-only sorption control. Experiments were run in triplicate, and errors are ± one standard deviation.

In the PPQ and CR sorption controls, the initial pH was 6.5 and after 31 days had buffered to approximately 7 and approximately 5, respectively (Figure S8g). This reflects the different sediment compositions and buffering capacities of the sediments, with the pH values bracketing the range of measured groundwater pH at the Sellafield site (see Section S1, Figure S2). In the sediment-only controls, removal of Sr^2+^ from the 1 mM groundwater solution occurred rapidly up to day 3, remaining constant thereafter. After 31 days, Sr^2+^ removal was 0.20 ± 0.06 mM (20%) for PPQ and 0.25 ± 0.04 mM (25%) for CR sorption controls (Figure 1). A similar trend was observed for the Ca^2+^ data, with initial rapid removal of sediment over the first 3 days (Figure 1a). The difference in uptake between the sediments is presumably due to the higher surface area of the CR sediment compared to PPQ.

#### Citrate Phosphate Amendments

**Figure 2:**
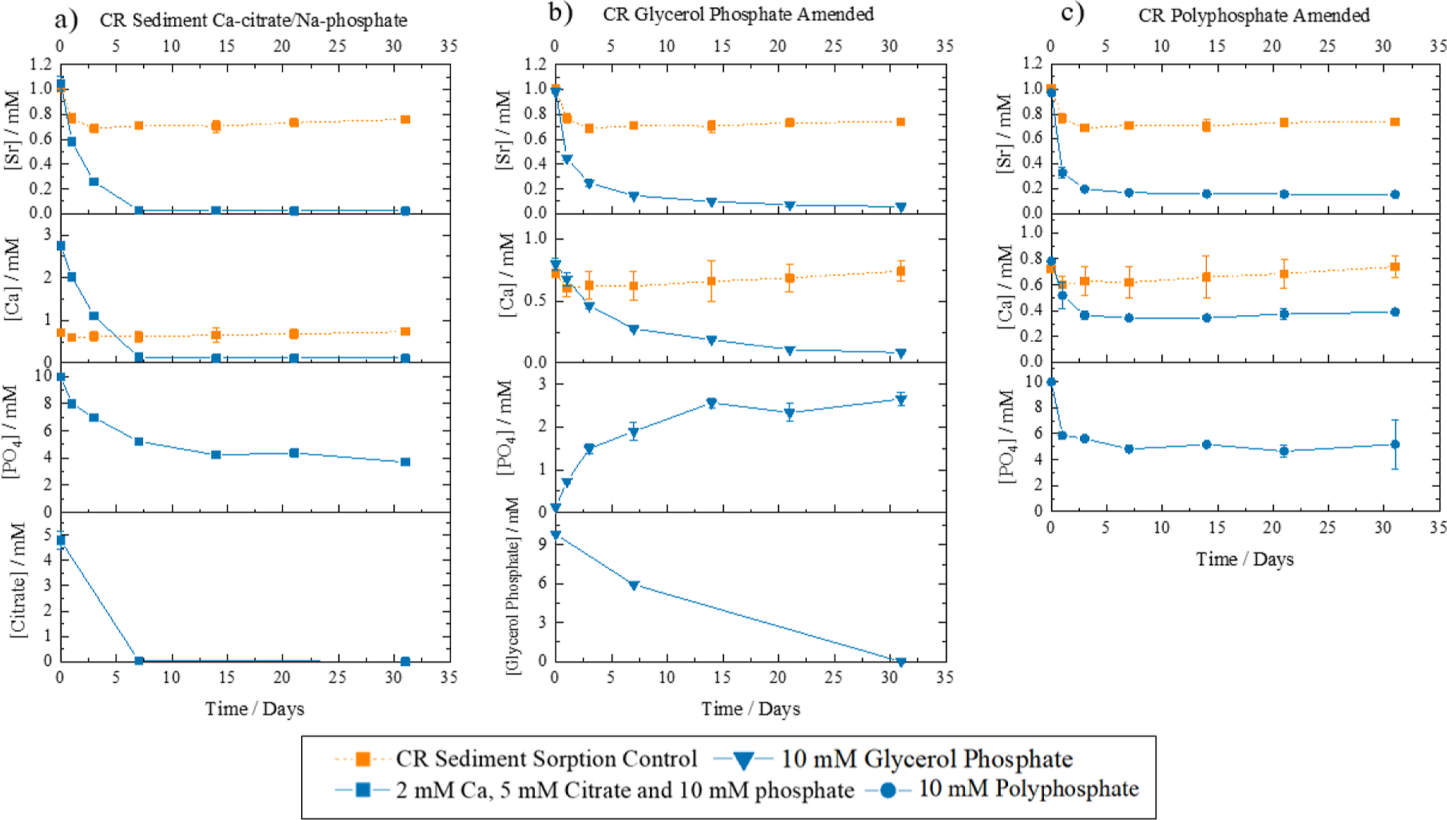
Aqueous geochemical data from CR sediment experiments amended with (a) Ca-citrate/Na-phosphate, (b) glycerol phosphate, and (c) polyphosphate and with the CR sediment sorption control plotted in each panel. Experiments were run in triplicate with error bars ± one standard deviation.

The addition of Ca-citrate/Na-phosphate amendments significantly enhanced the removal of Sr from the solution compared to the sorption controls [Figure 1 PPQ (a) and Figure 2 CR (a)]. In the PPQ sediment experiments, 0.97 mM (97%) Sr^2+^ removal was seen in the 1 mM Ca^2+^, 2.5 mM citrate, and 10 mM phosphate treatment after 31 days, which was mirrored by the Ca^2+^ removal. In addition, citrate was also completely degraded over 31 days. Finally, there was a continual decrease in the aqueous phosphate concentration from 10 to 6.4 mM throughout the experiment, and the pH increased from 6.5 to 8.5. In the higher concentration amendment (5 mM Ca^2+^, 12.5 mM citrate, and 10 mM phosphate), Sr^2+^ removal was less than the 1 mM Ca^2+^ amended experiment with 0.70 ± 0.07 mM (70%) removal from solution after 31 days. Here, the Sr^2+^, Ca^2+^, and phosphate concentration in the solution decreased quickly over the first 7 days and then removal was slower throughout the remainder of the experiment (Figure 1a). The co-removal of Sr^2+^, Ca^2+^, and phosphate over the first 7 days suggests that removal was initially dominated by calcium phosphate precipitation, with the longer time points plateauing, suggesting that the system may have reached equilibrium by 1 week. By contrast, in the 1 mM Ca^2+^, 2.5 mM citrate, and 10 mM phosphate experiment removal of Sr^2+^, Ca^2+^, and phosphate occurred throughout the 31 days of incubation. Here, after day 21 both Sr^2+^ and Ca^2+^ removal slowed, suggesting that the experiment was approaching equilibrium conditions despite the elevated concentration of aqueous phosphate (Figure S6d). The 1 mM Ca^2+^, 2.5 mM citrate, and 10 mM phosphate system showed complete removal of citrate by day 1 presumably driven by initial rapid sorption to sediment media and/or subsequent microbial degradation (Figure 1a).^63^ In contrast, at higher citrate concentrations (5 mM Ca^2+^, 12.5 mM citrate, and 10 mM phosphate system) the citrate data showed a more gradual removal throughout the experiment, with a fast initial removal likely due to citrate sorption to sediment followed by a slower removal by microbial degradation, with 32% of the initial citrate remaining in solution after 31 days. Ion chromatography analysis also showed an increase in acetate concentrations, a citrate breakdown product, throughout the course of the experiment in both the 1 mM Ca^2+^, 2.5 mM citrate, and 10 mM phosphate and 5 mM Ca^2+^, 12.5 mM citrate, and 10 mM phosphate PPQ systems (Figure S9). Additionally, in PPQ experiments, both of the Ca-citrate/Na-phosphate amendments showed an increased pH throughout the duration of the 31 days from pH 6.5 initially to pH 8.6 after 31 days (Figure S8a) when compared to the sorption control (pH 7.2 after 31 days) (Figure S8g). The modestly elevated pH in the citrate system was consistent with the consumption of H^+^ and production of carbonate ions during the aerobic breakdown of citrate by microbes within the sediments.^25,63,64^

In previous work on Hanford sediments, the effect of ionic strength on Sr^2+^ sorption to sediments was investigated during Ca-citrate/Na-phosphate amendment addition.^26^ Here, Sr^2+^ desorption from sediments was sensitive to the ionic strength of the initially injected amendments.^26^ Amendment solutions with elevated ionic strength showed a decreased uptake of Sr^2+^ to sediments. This effect likely explains the different uptake of Sr in the low (2.5 mM) and high (12.5 mM) citrate amendments in the PPQ experiments. The low amendment ionic strength (78 mM) was approximately half that of the high amendment (150 mM). Here, the increased ionic strength presumably leads to a decrease in sorption of Sr^2+^ on sediment during the initial injection of the solution due to Sr^2+^ cation exchange competition with Ca^2+^ and Na^+^.^17,26^ Additionally, the elevated ionic strength will inhibit Sr^2+^ sorption to newly precipitated biomineral phases, such as the Ca-phosphate phases expected in this experiment.^21^

To further explore likely strontium speciation in these experiments, thermodynamic modeling was conducted, using selected aqueous data from the experimental systems (Section S3). Here, in the 5 mM Ca^2+^, 12.5 mM citrate, and 10 mM phosphate experiment, the dominant molar Ca species throughout was predicted to be Ca-citrate (Ca(C_6_H_5_O_7_)^−^_(aq)_) (>80%) (Figure S6a). The presence of Ca-citrate presumably led to reduced Ca-phosphate precipitation due to the low activity of free Ca^2+^_(aq)_, as evidenced by the plateau in Sr^2+^, Ca^2+^, and phosphate concentrations after 7 days (Figure 1a). Indeed, modeling of the saturation indices (SIs) of the Ca-phosphate phase, Ca(HPO_4_)·2H_2_O (representative of amorphous Ca-phosphate phases as the precursor to more crystalline phases such as hydroxyapatite) showed that this phase was oversaturated from day 1 to 7. At this point, the calculated SI was at equilibrium from day 7 (Figure S6b). This was consistent with experimental results which show initial Ca removal and then a plateau from about a week and highlights the interplay between Ca-citrate complexation and precipitation and therefore Sr removal.

In contrast, modeling of Ca speciation in the 1 mM Ca^2+^, 2.5 mM citrate, and 10 mM phosphate experiment showed a change in percentage molar Ca speciation with time. Here, Ca-citrate was dominant (>80%) at the start of the experiment, and Ca^2+^_(aq)_ (31%) and Ca(HPO_4_)_(aq)_ (63%) were dominant after 1 day (Figure S6c) as the rapid removal of citrate from solution affected aqueous speciation. Modeling suggests that under low citrate conditions, free Ca^2+^_(aq)_ was available to react with aqueous phosphate, leading to Ca-phosphate oversaturation and precipitation. Further PHREEQC modeling (ThermoChimie (V10a) database^42^) with the chemical data for this system at longer time points predicted Ca(HPO_4_):2H_2_O oversaturation for the first 14 days of the experiment (Figure S6d), again broadly consistent with the experimental data, which show significant removal of Ca^2+^, Sr^2+^, and phosphate over the first 14 days (Figure 1a).

For the CR systems, the 2 mM Ca^2+^, 5 mM citrate, and 10 mM phosphate data were similar to the PPQ experiments. Here, there was significant removal of Sr^2+^ (97%), Ca^2+^ (96%), and citrate (100%) over 7 days compared to the sediment-only control which showed only 25% Sr removal after 31 days (Figure 2). The solution phosphate concentration also leveled off after 7 days, again implying precipitation of Ca-phosphate and concomitant Sr removal was dominating the system (Figure 2a). Both the CR sediment and parallel low-concentration PPQ sediment experiment showed almost total removal of Ca^2+^, Sr^2+^, and citrate. In contrast, the higher concentration PPQ experiment showed a lower total removal of Ca^2+^, Sr^2+^, and citrate, presumably due to higher ionic strength. This suggests that further optimization of concentrations of the Ca-citrate/Na-phosphate amendments may be possible.

#### Glycerol Phosphate Amendments

The addition of 10 mM glycerol phosphate significantly increased the removal of Sr^2+^ from the solution when compared to the sediment-only control (Figure 1b). In PPQ sediments, there was an increase in aqueous phosphate concentration from zero to 5 mM, during the first 21 days of the experiment. This coincided with a decrease in glycerol phosphate concentration from 10 to 0.5 mM likely as a result of aerobic microbial degradation of glycerol phosphate.^10,11,65^ After this, the phosphate remained at approximately 5 mM (Figure 1b).^10^ The increased phosphate in the solution was accompanied by a slow decrease in aqueous Sr^2+^ (60% removal) and Ca^2+^ (65% removal) over 21 days. After this, the Sr^2+^ and Ca^2+^ aqueous concentrations leveled off, consistent with the phosphate profile. This suggests precipitation of newly formed Ca-phosphate phases and resultant scavenging of Sr^2+^ was occurring during the early stages when free phosphate increased. The CR glycerol phosphate treatments showed increased Ca^2+^ and Sr^2+^ removal (90 and 94%, respectively) compared to parallel PPQ treatments (Figure 1b). Again, in the CR system, the phosphate concentration in the solution increased over 15 days before leveling off presumably as a result of Ca-phosphate precipitation. In both PPQ and CR glycerol phosphate experiments, the solution pH remained circumneutral (Figure S8c,d) throughout the experiment.

In systems amended with glycerol phosphate, there was a substantial variation in Sr removal between the two sediments used likely due to surface area effects and the differing microbial community within the two sediments (Figure S10). Here, both the PPQ and CR experiments showed similar glycerol phosphate degradation. Despite this, free phosphate and Ca^2+^ concentrations were lower in the CR system, which had a higher surface area. Regardless of these differences, the glycerol phosphate amendment caused enhanced removal of Sr (60% PPQ and 98% CR) when compared to the sediment-only control (20% PPQ and 25% CR) and the system may be further optimized by, e.g., addition of Ca^2+^ to the remediation treatment.

#### Polyphosphate Amendments

In the polyphosphate amended experiments, there was enhanced and rapid removal of Sr^2+^ in the PPQ experiment (55% Sr^2+^, 16% Ca^2+^, and 30% phosphate removal after 3 days), then solution concentrations leveled off. This compares to 20% Sr^2+^ removal in the sediment-only control (Figure 1c). In the polyphosphate experiment, equilibrium occurred after 3 days presumably due to the rapid formation of Ca-phosphates, and consistent with geochemical modeling which predicted a tendency for undersaturation of Ca-phosphates after 1 day (Section S3).

In the CR polyphosphate incubation, the same overall trend of enhanced removal of Sr^2+^ (70%), Ca^2+^ (35%), and phosphate (50%) (by day 3) compared to the sorption control (25% Sr removal) prior to leveling off was observed. Again, generally increased sorption in CR versus PPQ systems occurred (Figure 2c). Geochemical modeling of the solution data confirmed a trend toward undersaturation of Ca-phosphates after 1 day (Section S3). In both the PPQ and CR polyphosphate amended systems, pH remained circumneutral at 6.5–7 (Figure S8e,f) throughout the experiment. Again, Sr removal was enhanced in CR compared to PPQ systems suggesting the elevated surface area and indigenous microbial population in CR sediments may favor enhanced precipitation. Interestingly, in the presence of high surface area sediments (e.g., hydroxyapatite, Fe-oxides, and clays), adsorption of Ca^2+^, Sr^2+^, and phosphate may reduce the solution concentration of these species impacting Sr^2+^ fate, as observed for U systems.^6,66^

#### 16S rRNA Microbial Community Analysis

Analysis of the microbial community within the PPQ sediment at the start of the experiment, by 16S rRNA gene sequencing, showed a highly diverse community in the sediment (Figure S10). Approximately, half of the community (at genus level) was present at <1% relative abundance. The most abundant genus was *Perlucidibaca*, accounting for 28% of the community. This genus contains species such as *Phalaris aquatica*, a strict aerobe isolated from freshwater environments reflecting the sample location. The sediment-only control and polyphosphate-amended sediments after 31 days of treatment also showed diverse microbial communities. Here, both sediments showed a similar genus distribution, with the common soil bacteria *Achromobacter* (5.2% polyphosphate) and *Sedimentibacter* (7.0% sediment control) most abundant (Table S3). This suggests that the addition of polyphosphate did not lead to a pronounced enrichment of species within the sediment.

For the PPQ sediments amended with 1 mM Ca^2+^ and 2.5 mM citrate, and 5 mM Ca^2+^ and 12.5 mM citrate, and 10 mM glycerol phosphate, the microbial diversity after 31 days was reduced compared to the sediment-only control (Figure S10). For both citrate treatments, there was enrichment of bacteria from the genera *Sphingopyxis* (24.8%), *Pseudomonas* (12.9%), *Sedimentibacter* (7.2%), and *Janthinobacterium* (6.9%). These genera contain species capable of aerobic citrate assimilation including *Sphingopyxis macrogoltabida* and *Pseudomonas helmanticensis* and are commonly found in estuarine, marine, and soil environments.^67−70^ The enrichment of citrate-utilizing bacteria was consistent with citrate consumption and ingrowth of acetate (Figure S9) (citrate degradation product) observed in both citrate systems suggesting the microbial communities were degrading citrate in addition to sorption effects. After 31 days of glycerol phosphate amendment, the microbial community was enriched in bacteria from the genus *Pseudomonas*, *Ferruginibacter,* and *Cypionkella*, the latter two containing species with positive phosphatase activity (e.g., *Ferruginibacter profundus* and *Cypionkella psychrotolerans*).^71,72^ Enrichment for bacteria capable of phosphatase activity was consistent with the release of phosphate into solution within the glycerol phosphate amended system, via the cleavage of the C–PO_4_ bond (and utilization of glycerol as a carbon source).

The CR sediment initially showed a diverse microbial community with most genera detected at below <1% relative abundance (Figure S11). This large diversity was also seen in the sediments after 31 days of treatment with polyphosphate and in the sediment-only control, suggesting amending the sediment with polyphosphate did not enhance the microbial community, which was consistent with the PPQ sediment experiments.

By contrast, the CR sediment microbial community in the systems amended with 2 mM Ca^2+^ and 5 mM citrate, and 10 mM glycerol phosphate had a lower diversity compared to the sediment-only control. For the citrate amended system, there was a relative enrichment in species from the genus *Burkholderia*, *Chthoniobacter,* and *Geothrix* (Figure S11). These contain aerobic and facultative anaerobic species such as *Burkholderia ferrariae* which are common in soils and sediments. They also contain species such as *Geothrix fermentans* and *Chthoniobacter flavus,* which can ferment and assimilate citrate under both aerobic and anaerobic conditions (Table S3).^73−76^ The relative enrichment in citrate-degrading bacteria was consistent with the decrease in citrate seen within the citrate amended system and suggests microbial degradation of citrate is occurring in addition to sorption to sediment. For the CR glycerol phosphate experiment, there was enrichment in species from the genus *Novosphingobium*, *Azospirillum,* and *Curvibacter*, which contain aerobic and anaerobic species that have phosphatase activity.^77−80^ Enrichment of these species was consistent with the increase in aqueous phosphate as the bacteria capable of cleaving the C–PO_4_ bond in glycerol phosphate became enriched.

#### Sr Speciation and Solid-Phase Analysis

SEM–EDS of experimental end-point sediments was conducted to investigate the Sr^2+^ distribution and speciation. Here, for the PPQ sediments, the Ca-citrate/Na-phosphate (1 mM Ca^2+^, 2.5 mM citrate, and 10 mM phosphate), glycerol phosphate, and polyphosphate end point solid samples were imaged, and the CR sediments with 2 mM Ca^2+^, 5 mM citrate and 10 mM phosphate amendment were also imaged.

**Figure 3:**
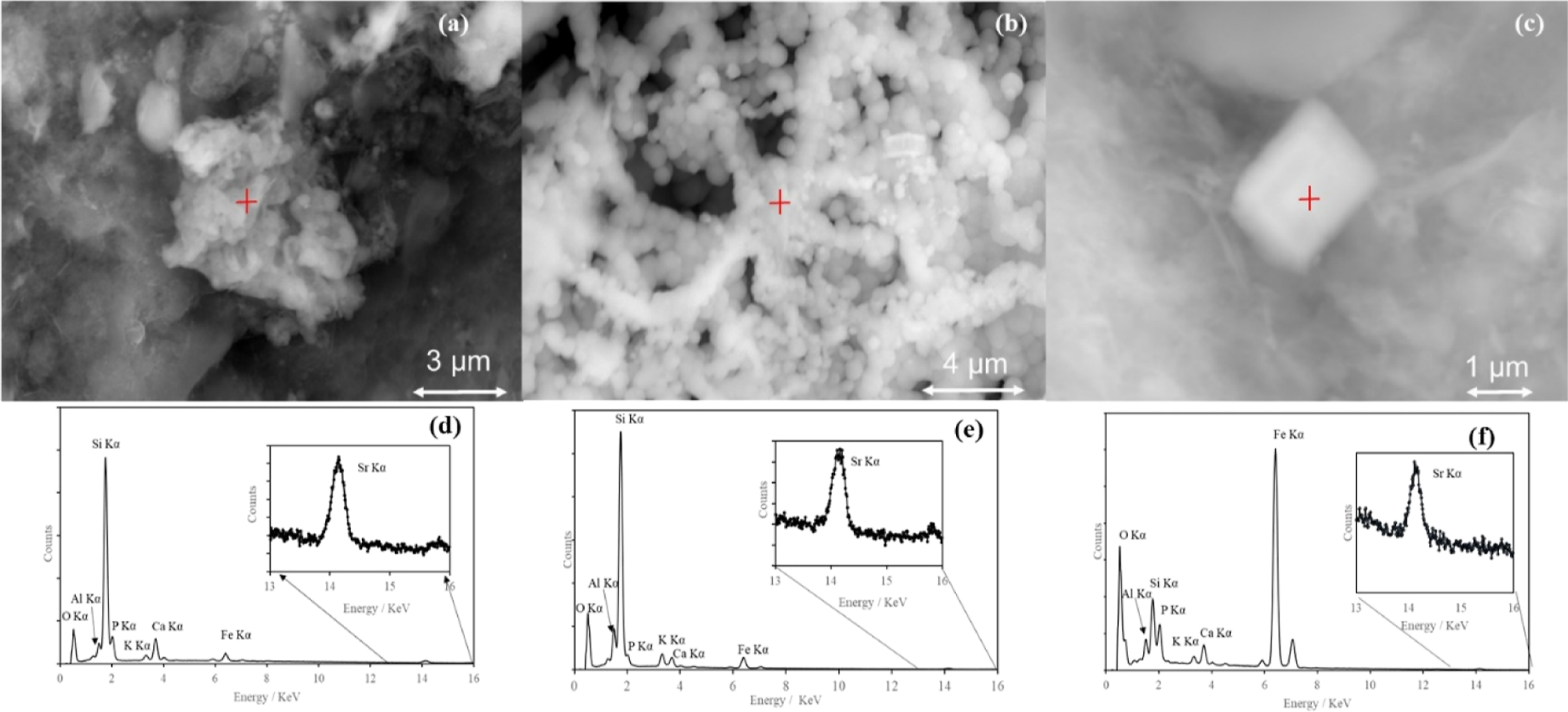
SEM secondary electron image of PPQ sediment after 31 days of amendment with (a) 1 mM Ca^2+^, 2.5 mM citrate, and 10 mM phosphate, (b) 10 mM glycerol phosphate, and (c) 10 mM polyphosphate, showing agglomerations of Ca, P, and Sr-containing submicron-sized particles. (d–f) Associated spot EDS spectra after 31 days of amendment with the red cross marking the analysis point.

For both sediments, glycerol phosphate- and Ca-citrate/Na-phosphate-amended experiments had spherical particle agglomerates (3–10 μm) (Figure 3a,b) consistent with bioprecipitated Ca-phosphate phases such as hydroxyapatite.^19,81^ Selected EDS and qualitative EDS mapping focused on these showed the co-location of Ca, P, and Sr peaks (Figure 3d,e) suggesting Ca-phosphate precipitation with associated Sr. Additional Si, Fe, and Al peaks in the (see Section S6) EDS spectra presumably relate to the quartz-/clay-rich sediment particles (see Section S6).

In the abiotic polyphosphate amended system, EDS–SEM analysis showed cuboid Ca-, P-, and Sr-rich particles (1–2 μm) on the sediment surface. These contrasted with the agglomerated clusters seen in the Ca-citrate/Na-phosphate and glycerol phosphate systems where phosphate cleavage was occurring biotically (Figure 3c). Interestingly, this suggests that biotic or abiotic precipitation impacted bulk Ca-phosphate morphology. Here, previous work on microbially mediated precipitation has shown similar agglomerated clusters.^19^ Co-location of Ca and P for the polyphosphate treatment was also observed along with background sediment Fe, O, Al, and Si peaks (see Section S6).

Across all three phosphate amendments SEM–EDX confirmed Sr^2+^ was co-located with Ca-phosphate phases, this indicates that Sr^2+^ was sequestered from solution through sorption/co-precipitation to these Ca-phosphate phases. To further understand these associations XAS analysis was undertaken.

#### X-ray Absorption Spectroscopy

**Figure 4:**
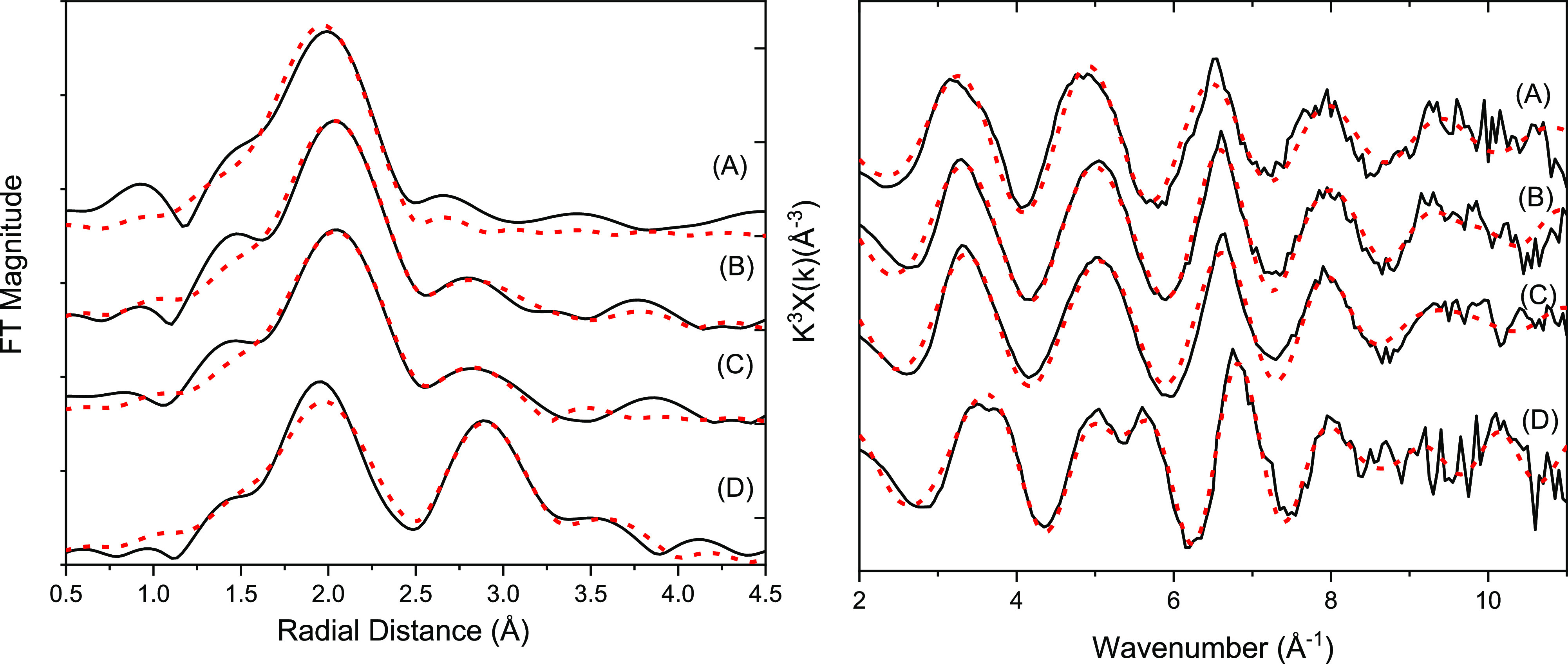
Experimental Sr K-edge EXAFS data collected from PPQ sediments after 31 days of treatment with (A) sorption control, (B) Ca-citrate/Na-phosphate, and (C) glycerol phosphate. (D) 4% Sr doped hydroxyapatite standard. Data, black line; theoretical best fit, red dashed line.

Sr K-edge XAS was conducted on selected samples (Figure 4). EXAFS data were collected on PPQ sediments after 31 days on a Sr^2+^ sorption control, as well as the low Ca-citrate/Na-phosphate (1 mM Ca^2+^, 2.5 mM citrate, and 10 mM phosphate), and glycerol phosphate amendments (Figure 4 and Section S7). The PPQ sorption control was the best fit, with 9 oxygen atoms at 2.60 Å consistent with the outer-sphere adsorption of Sr to the sediment.^17,82^ Indeed, it was not possible to fit additional shells of backscatters beyond 3.0 Å, confirming Sr^2+^ was predominantly adsorbed to the sediment as an outer sphere complex.^8,17^

In the Ca-citrate/Na-phosphate amended system, 9 oxygen atoms were fitted at 2.61 Å; however, fitting of additional shells beyond 3.0 Å was necessary to resolve the spectral features suggesting some incorporation. Informed by the relevant literature and assuming a hydroxyapatite-like local coordination environment, a shell was fitted with 2 P atoms at 3.28 Å, consistent with Sr–P distance (3.25 Å) in the Sr-incorporated hydroxyapatite standard and in past works.^34,83^ The best fit Sr–P shell occupancy of 2 is lower than the expected value of 3 in fully incorporated hydroxyapatite,^34,83^ suggesting that incorporation of up to approximately 66% Sr^2+^ into the Ca-phosphate phase was occurring.^8^ In addition, for both the Ca-citrate and glycerol phosphate Sr EXAFS analysis, it was not possible to fit a second P shell at ∼3.6 Å. This shell is clearly present in the Sr-incorporated hydroxyapatite standard, and as Sr is not modeled in this coordination environment, this suggests that Sr removal is occurring through both sorption and (only partial) incorporation into a poorly ordered hydroxyapatite-like phase. The lower coordination numbers observed in our EXAFS fits relative to the crystalline hydroxyapatite standard reflect the poorly ordered nature of the newly formed Ca-phosphate biominerals. This is consistent with past work, which shows that in the citrate-phosphate amendment, poorly ordered Ca-phosphate phases form initially and age to more crystalline, hydroxyapatite-like phases.^4,26^

Essentially the same fit was used for the glycerol phosphate amended system with the best fit with 9 oxygen atoms at 2.60 Å and 2 P atoms at 3.27 Å. It was possible to fit a statistically significant (95% *f*-test) third shell of Ca atoms at approximately 4.1 Å (Table S4), consistent with the Sr–Ca distance in the Sr-incorporated hydroxyapatite standard (4.05 Å) and reported in the literature (4.02–4.15 Å).^8,34,83−85^ By contrast, the addition of a third Ca shell in the Ca-citrate/Na-phosphate sample gave an *f*-test value of 66%, suggesting less strong evidence for Ca-backscatterers in these data.

Overall, the XAS data suggest that in contrast to the sorption control, Sr^2+^ was significantly (up to 66%) incorporated into a poorly ordered Ca-phosphate phase with a hydroxyapatite-like local coordination environment, in both the Ca-citrate/Na-phosphate and glycerol phosphate amended microcosms, which is consistent with past work.^8,26^ Residual, non-incorporated Sr^2+^ is presumably sorbed to the mineral phases in the sediment including Ca-phosphates. Interestingly, field trials at Hanford, USA using Ca-citrate/Na-phosphate amendments have shown continued incorporation of sorbed ^90^Sr into Ca-phosphates over extended periods of months to years after initial Ca-citrate/Na-phosphate injection.^4,26^ This suggests that aging of Sr^2+^ sorbed to Ca-phosphate may further increase Sr incorporation into the Ca-phosphate mineral phases as recrystallization to hydroxyapatite-like phases occurs.

#### Environmental Implications

All three of the in situ amendments enhanced the removal of Sr^2+^ from the solution when compared to sediment-only systems. Interestingly, there was significant variation in removal between the treatments despite all three amendments producing Ca-phosphate phases. The removal of aqueous cations by Ca-phosphate precipitation occurs via a variety of processes (e.g., adsorption, ion exchange, surface complexation, co-precipitation, and recrystallization) all of which are dependent on the biogeochemical conditions in the experiment.^86^ One of the key factors controlling the differences in Sr^2+^ removal across the three amendments is the amount of Ca-phosphate produced. Here, the biotic systems (Ca-citrate/Na-phosphate and glycerol phosphate) showed enhanced removal of Ca and phosphate over extended times compared to the abiotic (polyphosphate) treatment, suggesting elevated Ca-phosphate precipitation, which led to greater Sr^2+^ removal through sorption and incorporation into the Ca-phosphate phases. Additionally, the gradual precipitation of Ca-phosphate observed throughout the biotic experiments leads to the continuous production of new surface sites for Sr^2+^ sorption and subsequent incorporation.^19^ Once precipitated, Ca-phosphate phases will adsorb Sr^2+^, the amount of which is largely controlled by solution pH and ionic strength, with the point of zero charge (pH_pzc_) for hydroxyapatite being 7.5.^86^ In the Ca-citrate/Na-phosphate and glycerol phosphate amended systems (biotic) the pH increased from 6.5 to 8.5 and 7.6, respectively, which enhanced cation sorption to the precipitating Ca-phosphates, as above the pH_pzc,_ the surface will possess an increased negative charge thus attracting Sr^2+^. This increase in solution pH was not seen within the abiotic (polyphosphate) system (pH 6.7); thus, any precipitated Ca-phosphates will have a more positively charged surface (pH < pH_Pzc_), which would result in lower rates of removal. Although the expected concentrations of ^90^Sr in Sellafield-contaminated groundwaters are orders of magnitude lower than stable Sr, past work has shown that ^90^Sr behaves comparably to stable Sr.^25,63^ Additionally, the higher solid/solution ratio expected in deployment scenarios in the subsurface will provide additional sorption sites, and biostimulation is intended to increase the rate of Ca-phosphate precipitation. Additionally, the degree of mixing between groundwater sediments and injected amendment solutions will be reduced compared to microcosm experiments, and this aspect clearly warrants further investigation via, e.g., column and field experiments.

The removal of Sr^2+^ (97% in PPQ and CR) and citrate from solution in the Ca-citrate/Na-phosphate amended systems was consistent with previous work carried out at the Hanford nuclear site. Here, significant (>95%) removal of ^90^Sr from groundwater plumes was seen at laboratory and field scale.^4^ This suggests that scale-up of the Ca-citrate/Na-phosphate may deliver similar remediation potential for ^90^Sr-contaminated plumes across nuclear sites, including Sellafield. The CR glycerol phosphate amended systems also showed Sr^2+^ removal similar to that of previous work using Calder River sediments.^8^ However, removal was lower in the PPQ sediment system (60% compared to 95% in the previous work using CR), likely reflecting the lower surface area of the PPQ sediment, and the differing microbial communities and geochemical conditions present in the two systems.^8^ Here, exploration of Ca^2+^ amendments to optimize Ca-phosphate precipitation in the glycerol phosphate was not explored, and the Ca^2+^ concentration was lower than that for the citrate amended experiments. Past work on the abiotic polyphosphate amendment was focused on U sequestration from groundwaters via the formation of insoluble U(VI) phosphate phases.^32,87^ Our work in batch experiments suggests this abiotic technique is applicable to remediation of ^90^Sr-contaminated Sellafield groundwaters but is less effective than either of the biotic treatments.

In situ deployment of these phosphate generating techniques aims to produce Ca-phosphate phases such as hydroxyapatite, with removal of ^90^Sr occurring through sorption and incorporation into phosphate phases. Ca-phosphate phases are expected to be recalcitrant to re-dissolution under environmental conditions and once incorporated, ^90^Sr can undergo radioactive decay, with reduced risk of remobilization. Given the 30 year half-life of ^90^Sr, significant radioactive decay of ^90^Sr will have occurred over 120 years, which are the current decommissioning times for many nuclear sites, including Sellafield.^88^

## Conclusions

Three different slow-release phosphate amendment systems were shown to generate Sr-containing Ca-phosphate minerals in aerobic microcosms under representative Sellafield conditions. Here, microbially mediated Ca-phosphate mineralization was stimulated using Ca-citrate/Na-phosphate and glycerol phosphate amendments, with the abiotic polyphosphate treatment leading to direct precipitation. Enhanced Sr^2+^ removal from the solution was observed in all three treatments compared to the sediment-only sorption control. Overall, Sr^2+^ removal was enhanced in the order Ca-citrate/Na-phosphate (97%), glycerol phosphate (60%), and polyphosphate (55%), providing a positive prospect for these remediation approaches to be applied in a range of nuclear sites.

Interestingly, the 1 mM Ca^2+^, 2.5 mM citrate, and 10 mM phosphate Ca-citrate/Na-phosphate amendments were the best at removing Sr^2+^ in the microcosm systems with 97% removal in both PPQ and CR. Characterization of treated sediments confirmed the formation of Ca-phosphates and highlighted evidence both for sorption of Sr^2+^ to Ca-phosphates and for partial incorporation of Sr^2+^ in all three treatments. The high removal levels seen in the Ca-citrate/Na-phosphate amended systems in both PPQ and CR sediments suggest that it would perform well in the heterogeneous subsurface at Sellafield. Before prioritizing this treatment, it is beneficial to consider further work on these systems, e.g., whether Ca^2+^ co-amendments could improve the performance of glycerol phosphate systems.

## Supporting Information Available

Benchtop study of the Sellafield groundwater, sediment XRD and XRF analysis, geochemical modeling, microcosm pH and IC measurement, 16S rRNA microbial community analysis, SEM imaging and EDS mapping, and XAS analysis and EXAFS fitting (PDF)
