# Supplementary material for: Phosphate (Bio)mineralization Remediation of 90Sr-Contaminated Groundwaters
Source: ACS ES T Water. 2023 Aug 31;3(10):3223–34. doi: 10.1021/acsestwater.3c00159 (PMC10580321; doi:10.1021/acsestwater.3c00159)
Supplement: Supplementary file 1 — ew3c00159_si_001.pdf [file ew3c00159_si_001.pdf]

Supporting Information

Phosphate (Bio)mineralisation Remediation of ^{90}Sr Contaminated Groundwaters

Callum Robinson,[1] Samuel Shaw,[1] Jonathan R. Lloyd, [1] James Graham, [2] Katherine Morris [1]*

[1] Research Centre for Radwaste Disposal and Williamson Research Centre for Molecular Environmental Science, Department of Earth and Environmental Sciences, The University of Manchester, Manchester, M13 9PL, U.K.

[2]National Nuclear Laboratory, Sellafield , Cumbria, CA20 1PG, U.K..

This document provides supporting information for this paper, including a bench top study of the Sellafield groundwater, sediment XRD and XRF analysis, geochemical modeling, microcosm pH and IC measurement, 16S rRNA microbially community analysis, SEM Imaging and EDS mapping and XAS analysis and EXAFS fitting.

Contents

Section S1: Sellafield Groundwater Study 2
Section S2: XRD and XRF Analysis 11
Section S3: Geochemical Modeling 17
Section S4: pH Measurement and IC analysis from Microcosm Experiments 20
Section S5: 16S rRNA Microbial Community Analysis 22
Section S6: SEM/EDS Spot Images and EDS Elemental Mapping 32
Section S7: XAS Fitting Parameters 39

Section S1: Sellafield Groundwater Study

Abstract. A bench top groundwater study was conducted using borehole data from the Sellafield nuclear site, UK to develop a new synthetic groundwater media, for use in experimental systems simulating the subsurface conditions beneath Sellafield. Specific focus of the study was given to the composition within strontium 90 contaminated groundwater plumes, which have arisen from historical activities at Sellafield. Direct access to on-site borehole data provided us with an opportunity to improve on previous groundwater media and develop an up to date groundwater methodology with a clear and traceable origin. We suggest a method for the synthesis of this synthetic groundwater media and provide discussion as to the origin of the mixed type assemblage classification given to the plumes groundwater.

Introduction. Experiments attempting to simulate the subsurface conditions below the Sellafield nuclear site, have previously used a synthetic ground water media developed by (Wilkins et al., 2007) to represent the subsurface groundwater conditions at Sellafield. This media was developed for use in experimental systems investigating biogeochemical process occurring beneath the Low-Level Waste Repository (LLWR) at Drigg, UK.[1] Although this site is close to the Sellafield estate the groundwater water composition is heavily altered by anthropological effect, predominantly the leaching of cementitious material from concrete lined ditches and the large volume of cement LLW waste containers. This resulted in a calcium bicarbonate dominated groundwater media that had higher concentrations of carbonate, calcium and silicates than is typically found in the groundwaters beneath the Sellafield estate.[2]

Distributed across the Sellafield estate are approximately 150 boreholes which are used to sample the groundwater beneath the site. Subsequent analysis of these samples provides information on the concentrations of radionuclides and the major cations/anions that make up the groundwater composition at a given borehole. Campaigns of sampling occur at several times per year, allowing Sellafield to determine the movement and distribution of radionuclides within the subsurface. In addition to providing valuable information as to the groundwater composition beneath the Sellafield site. It is with these periodic reviews of groundwater data that Sellafield can track the movement of contamination plumes away from there source. This review is predominantly concerned with the groundwater composition within the path of the ^{90}Sr plumes that originate from the legacy facilities within separation area.

In the 2016 Sellafield groundwater review, the distribution of groundwater types across the Sellafield estate is reported, with a majority being assigned mixed type groundwaters with either bicarbonate, calcium, chloride, sodium or bicarbonate, calcium, chloride, magnesium, sodium, sulphate types. There is evidence of some calcium bicarbonate dominated groundwater types, however these are limited to boreholes close to the site boundary and therefor do not reflect the groundwater regime within the separations area of site or within the path of the ^{90}Sr plumes.

Methodology. pH measurement, concentrations of the major cations (Ca^{2+}, Na^{+}, Mg^{2+}, K^{+}), anions (HCO_3^-, Cl^-, NO_3^-, SO_4^{2-}), strontium-90 (bq/l) and some minor ions (NH_4^+, NO_2^-,$CO^{2-}{}_3$) within each borehole from the 2006 to 2019 were provided by the National Nuclear Laboratory (NNL). Once the data had been sourced the borehole location was overlaid atop the concentrations of ^{90}Sr within the subsurface to determine which would best describe the groundwater composition within the ^{90}Sr plumes. 65 boreholes within the path of the Sr plumes were chosen.

Each sample point within the lifetime of the borehole was charge balanced using the concentrations of the major ions and an acceptance condition of ± 5% was introduced. Although not typically defined as a major ion during groundwater studies the concentration of nitrate was included within these calculations as it is present at significantly high concentrations within the Sellafield groundwater (6-200ppm). The minor ions (NH_4^+, NO_2^-,$CO^{2-}{}_3$) were not used in subsequent calculations as the data was of poor quality, with only sporadic reporting of concentrations and a low confidence in the reliability of the data that was reported due to incorrect recurring values persisting over many years.

Data points that did not meet this ± 5% criterion were rejected. The concentrations of the major cations (Ca^{2+}, Na^+, Mg^{2+}, K^+) and anions (Cl^-, HCO_3^-, NO_3^-, SO_4^{2-}) for each charge balanced sample point were plotted in a series of histograms and an average for each computed. To ensure this data was representative of the average groundwater composition within the ^{90}Sr plumes, sample points that exceeded ±5σ (standard deviations) values from the mean were emitted. As data points above 5σ are likely results of contamination events or errors in measurement and not representative of the average groundwater composition. The ingress of salt water from the Irish Sea had a noticeable effect on borehole samples from the south of site, with elevated Na^+ and Cl^- concentrations several times that of borehole samples from the north of site. To ensure this did not affect the groundwater study, any suspected of having significant saltwater ingress (Na^+ or Cl^- elevated above 4σ from the mean) were rejected from the study.

This left 46 boreholes and 344 sample points as the basis for constructing the new groundwater composition. Not all these data points contained a pH measurement and therefor the distribution of groundwater pH was calculated using the 192 sample points (39 boreholes) that did provide pH data. Only readings within the pH range 4.75-8.5 were considered, as it is likely readings outside

this range are due to contamination events or errors in the measurement as previous studies have recorded the pH range of the Sellafield site between pH 4.75-8.33 [2] reducing the total sample number to 183. The average groundwater pH of the ^{90}Sr plume was calculated by computing the arithmetic average of the 183 pH values supplied. It must be noted however, that this value does not represent the true average pH of the plume, as pH data is logarithmic attempting to average data points ranging over multiple pH units (4-8.5) would be improbable, as there are clearly differing domains of acidity/alkalinity within the subsurface. An attempt to calculate an average pH value was done as a first approximation.

Results and Discussion. The histograms shown in figure S1, depict the distribution in concentrations of each major cation and anion from the 46 boreholes. The majority of which are pseudo normally distributed about a mean value. This provides confidence that the critical analysis carried out during the groundwater study has produced a reliable and accurate average groundwater composition.

A groundwater type is defined by the dominant cation and anion species dissolved within a groundwater sample. The equivalence concentration of the cation or anion must be greater than 50% of the total cation or anion equivalence to be defined as dominant, if not the groundwater is assigned a mixed type, and the ions are listed in decreasing order of dominance. For the ^{90}Sr plume groundwater the type was calculated as mixed, with the most dominant cations being (% total cation equivalence) Na^+ 44%, Ca^{2+} 40%, Mg^{2+} 14% and K^+ 2% and anions (% total anion equivalence) Cl^- 45%, HCO_3^- 29%, SO_4^{2-} 16%, NO_3^- 10%. The binomial distribution of the bicarbonate concentration could be a reflection in the differing groundwater domains within separation area and the South of site. Boreholes were selected from across the path of the ^{90}Sr plumes (separation area and the south of site) and the data likely reflects the differences in

groundwater properties of these two regions of site. A result that is shown in the 2016 groundwater report, [2] with boreholes within separation area characterised as mixed groundwater types, containing predominantly HCO_3^-, Ca^{2+},Cl^- and Na^+ ions, and boreholes on the South of site being characterised as sodium-chloride types, due to a dominance of Na^+ and Cl^- ions within the groundwater. This difference is likely due to the effects of saltwater ingress from the Irish Sea [2] and from anthropological effect, predominantly the leaching of cementitious material from concrete lined silos and buildings within the separations area increasing dominance of bicarbonate and calcium ions within the groundwater.

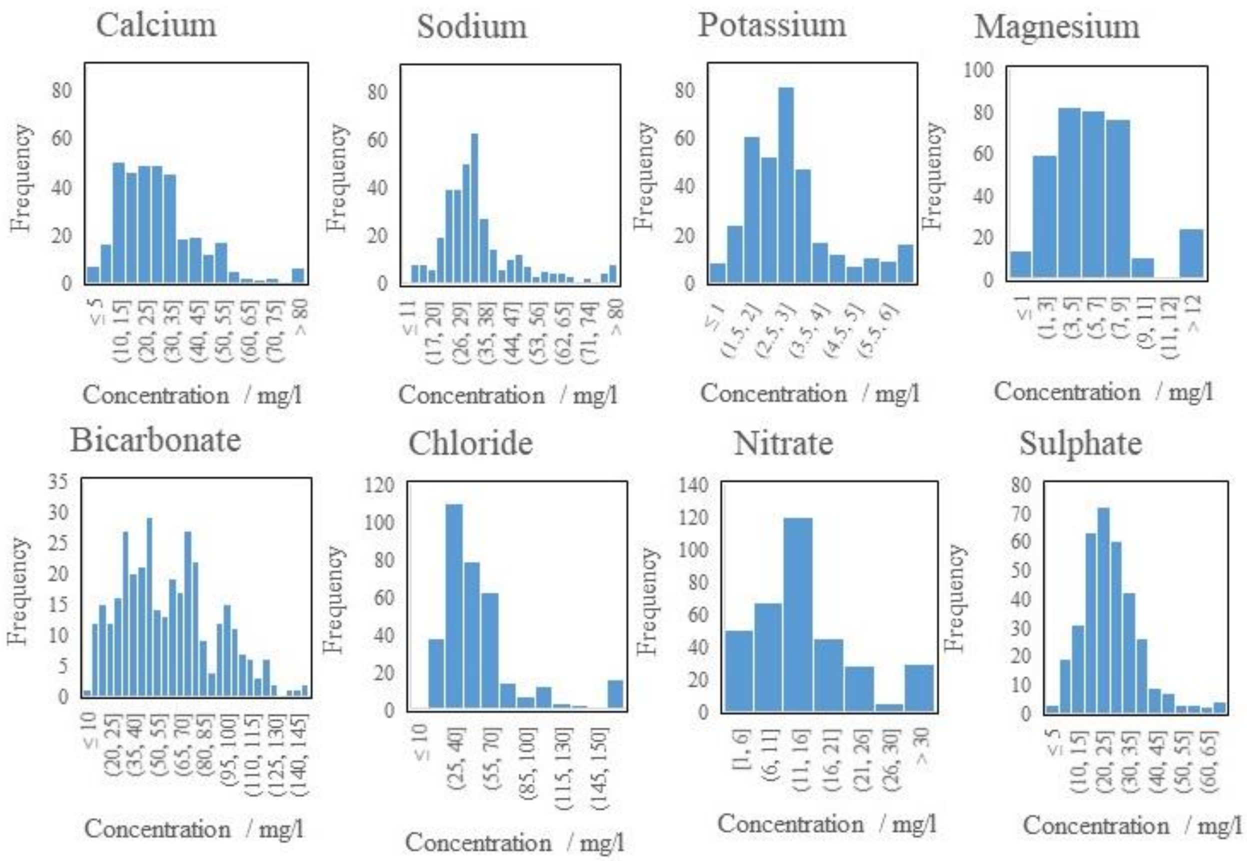

Figure S1. Distribution of the major cations and anions within Sellafield ^{90}Sr plume groundwater.

The average ^{90}Sr plume groundwater pH (6.51), with figure S2 showing the distribution in pH values from each sample point used.

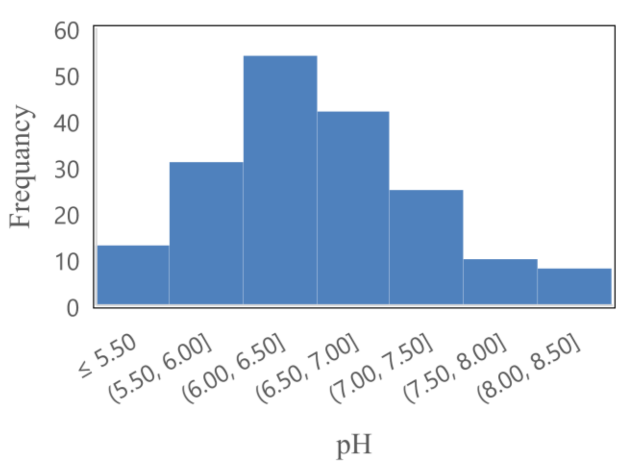

Figure S2. Distribution of groundwater pH, within Sellafield ^{90}Sr plume groundwater.

This analysis produced a new groundwater composition that was representative of the environment within the ^{90}Sr plumes as shown in Table S1 below:

Table S1. Average concentration of the major cations and anions within the groundwater at the ^{90}Sr plume at Sellafield.

Sellafield ^{90}Sr Plume Groundwater Composition			
Ion	meq /L	ppm	mM
Ca^{2+}	1.38	27.73	0.69
Mg^{2+}	0.48	5.82	0.24
K^{+}	0.07	2.87	0.07
Na^{+}	1.53	35.19	1.53
HCO_3^-	0.98	60.03	0.98
Cl^-	1.52	53.79	1.51
NO_3^-	0.32	19.98	0.32
SO_4^{2-}	0.52	25.18	0.26

The groundwater composition of the ^{90}Sr plumes was used as a basis for designing a new Synthetic GroundWater (SGW) media. Table S2 gives a summary of the proposed new media.

Table S2. Synthetic groundwater recipe for Sellafield representative groundwater.

New Media		
Salt Dissolution	mM	mg/L
$MgSO_4.7H_20$	0.20	49.54
$CaSO_4$	0.07	9.53
KCl	0.07	5.22
NaCl	0.20	11.69
$CaCl_2. 2H_20$	0.62	91.15
$NaNO_3$	0.32	27.20
$NaHCO_3$	0.98	82.33

The new SGW charge balance was calculated at +1.78%, well within the ±5% criteria used to critically assess the Sellafield data. Confirmation as to the long-term stability of SGW was achieved by experimental observation. With no visible formation of solid phases several weeks after synthesis in both systems open and closed at atmosphere across varying pH (5-8). Additional long term stability confidence was confirmed using geochemical modelling (PHREEQC),[3] with no precipitation predicted. Demonstrating the stability of the solution across environmental conditions relevant to that of the Sellafield site.

Conclusion. A bench top groundwater study was conducted to critically assess the groundwater composition of ^{90}Sr plumes within the Sellafield site. With access to borehole data from the previous 15 years of the Sellafield subsurface we have produced an average groundwater composition which reflects the differing domains present in the Sellafield subsurface, whilst also reflecting the average composition within the ^{90}Sr plumes. This work provides a foundation for the synthesis of a synthetic groundwater media that will be used experimental systems that aim to simulate the Sellafield subsurface.

Section S2: XRD and XRF Analysis

Additional characterization data for Peel Place Quarry (PPQ) sediment can be found from (Purkis and Robinson et al, in preparation). CR sediments were previously characterized by Law et al 2010. [4] Sr doped hydroxyapatite standard was synthesized following the methods of Afsha et al 2003 and Catros et al 2010 [5,6], using $SrCl_2$ to achieve a 4% substitution as confirmed by XRD.

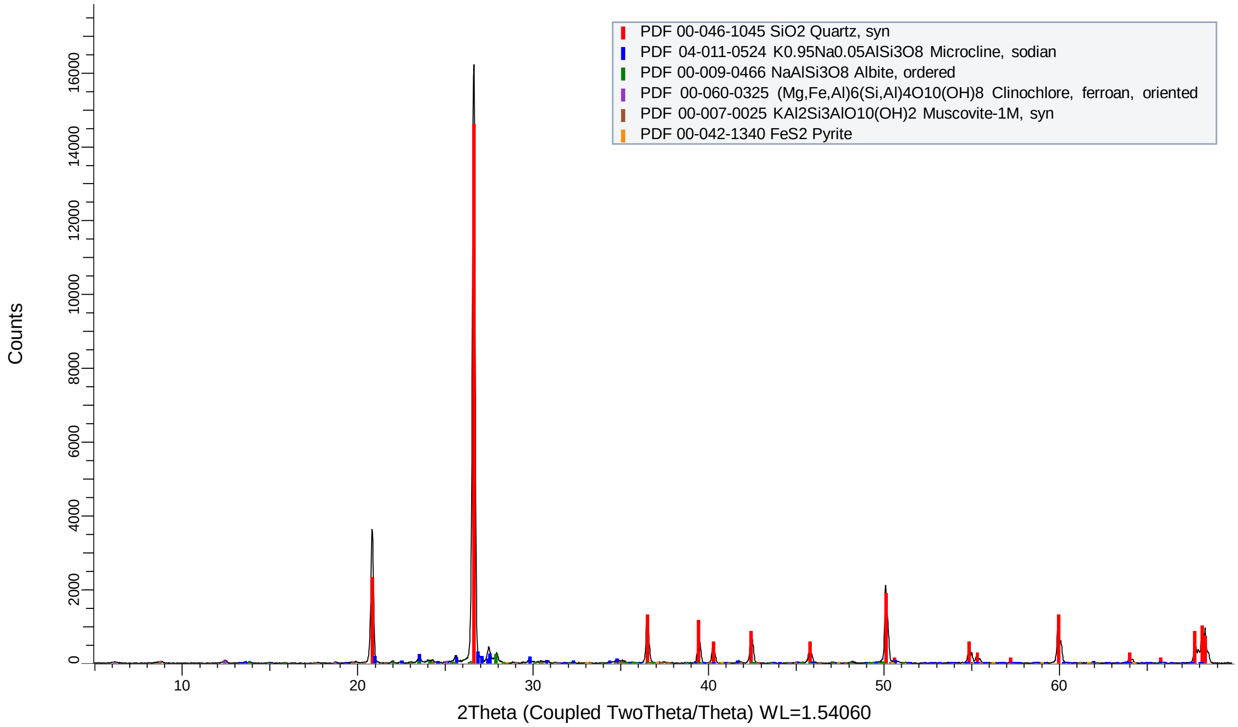

Figure S3. Peel place quarry sediment XRD pattern.

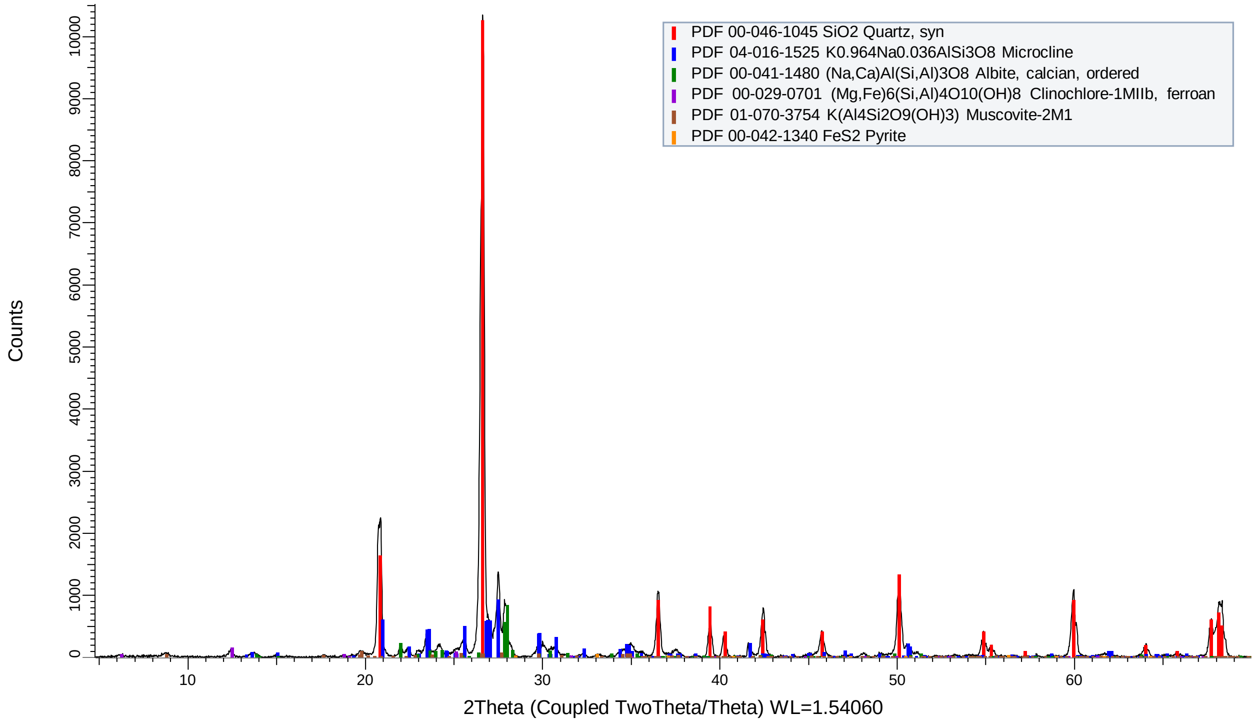

Figure S4. Calder River sediment XRD pattern

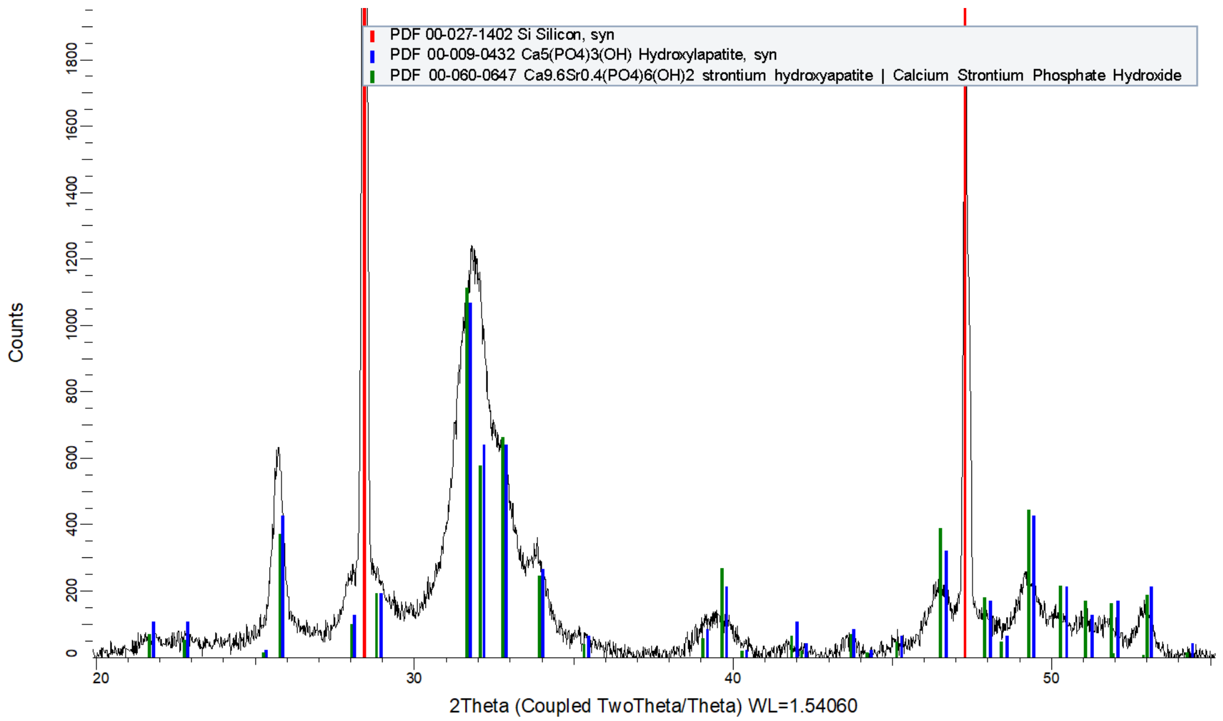

Figure S5. Sr doped Hydroxyapatite standard.

Table S3. XRF results for major and trace element composition of PPQ sediment.

Majors	Concentration / %
Na_2O	0.6
Al_2O_3	4.3
SiO_2	90.4
P_2O_5	0.1
SO_3	<0.1
Cl	<0.1
K_2O	1.9
CaO	0.2
TiO_2	0.2
Fe_2O_3	1.4
MgO	0.7

Trace	Concentration / ppm	
**Element**	**PPQ Sediment**	**LLD**
Sc	2.2	1
V	16.5	1.1
Cr	25.5	0.9
Mn	258	1.5
Co	66.7	1.5
Ni	8	0.4
Cu	5.3	0.5
Zn	13.6	0.4
Ga	4.2	0.3

Ge	2.7	0.3
As	53.7	4.3
Rb	43.8	0.3
Sr	64.2	0.2
Y	6.9	0.3
Zr	113.2	0.4
Nb	2.9	0.3
Sn	1.7	2.1
Te	47.4	2.6
Cs	3.7	2.3
La	5.7	5.2
Ce	10.5	6.9
Nd	8.3	3.5
Sm	0	3.2
Yb	0.5	1.4
Hf	1.8	2
Hg	7.8	3.2
Th	2.2	1

Section S3: Geochemical Modeling

Geochemical modelling was conducted using experimental aqueous data to determine the aqueous Ca speciation in the PPQ sediment Ca-citrate/Na-phosphate amended systems and the saturation index of amorphous calcium phosphate phases within PPQ and CR polyphosphate experiments. These amorphous phases are the first to precipitate and are precursors to more crystalline phases such as hydroxyapatite. All calculations employed the PHREEQC version 3 geochemical modelling program,[3] using aqueous data from experimental systems with the ThermoChimie (V10a) database.[7]

Ca-citrate/Na phosphate amendment

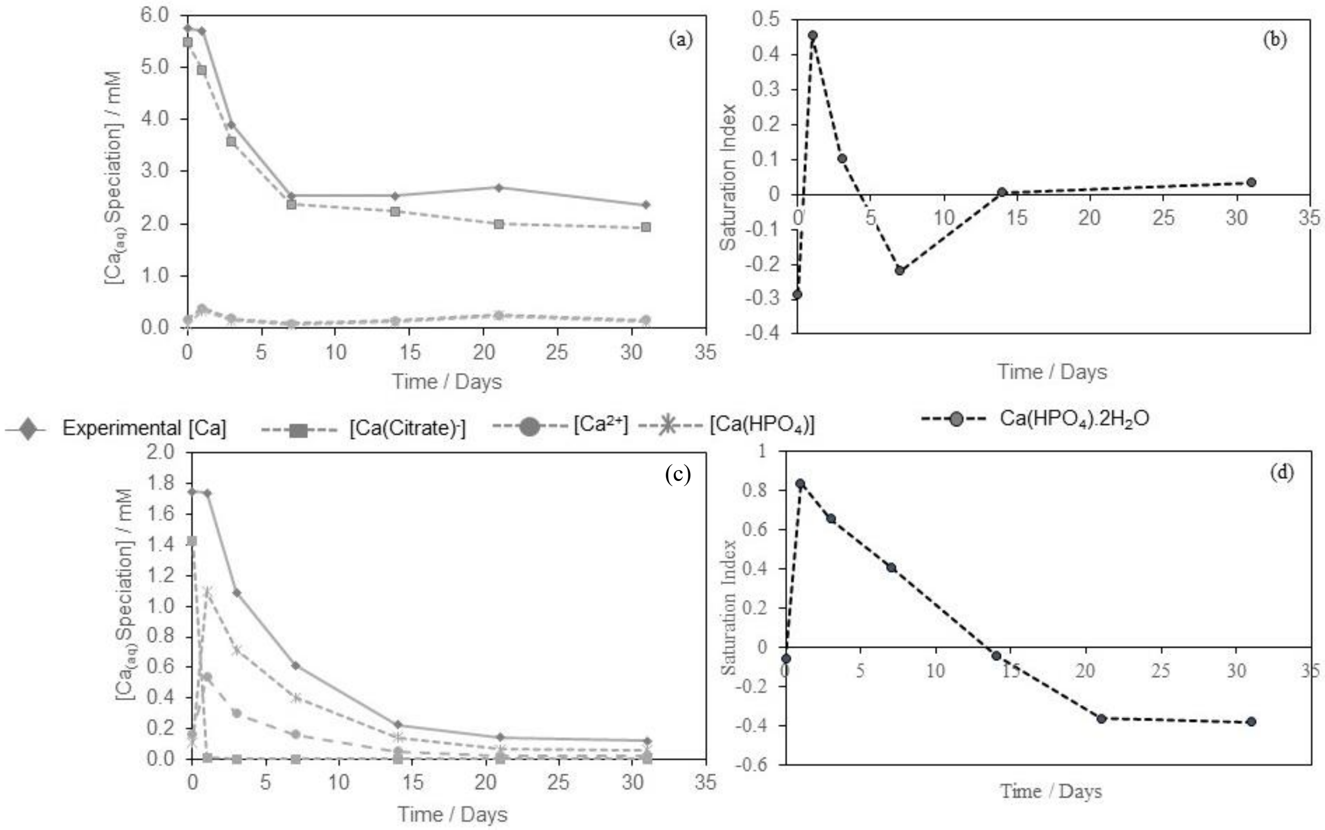

Figure S6. Geochemical model of the Ca speciation in the 5 mM Ca^{2+}, 12.5 mM citrate, 10 mM phosphate (a) 1 mM Ca^{2+}, 2.5 mM citrate and 10 mM phosphate (c) amended PPQ sediment experiments. With the saturation index for $Ca(HPO_4).2H_2O$, shown for the 5 mM Ca^{2+}, 12.5 mM citrate, 10 mM phosphate (b) and 1 mM Ca^{2+}, 2.5 mM citrate and 10 mM phosphate (d) concentration system .

Polyphosphate amendment

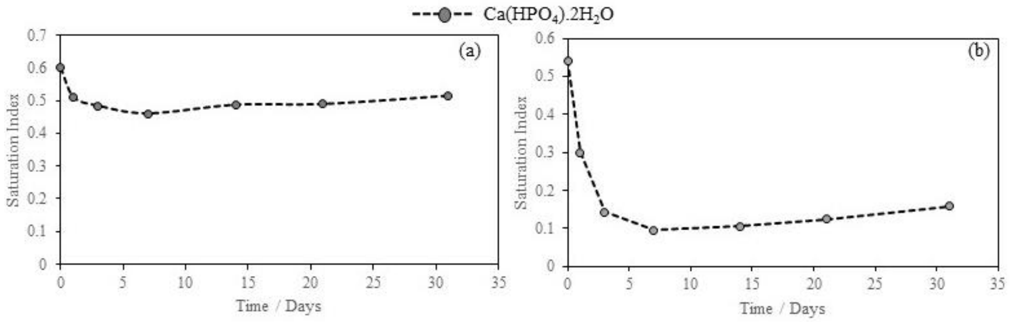

Figure S7. Geochemical model of the saturation index for $Ca(HPO_4).2H_2O$, for PPQ sediment (a) and CR (b) sediment experiments amended with 10 mM polyphosphate.

Section S4: pH Measurement and IC analysis from Microcosm Experiments

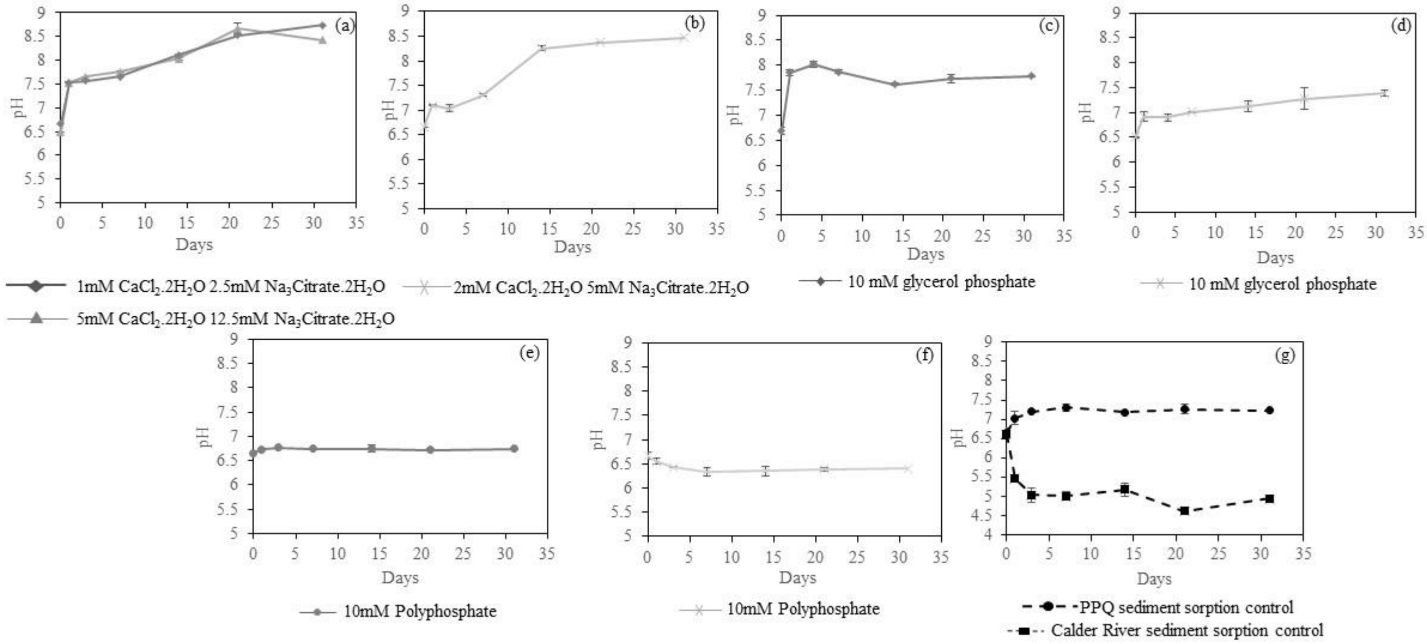

Figure S8. pH measurements from phosphate mineralisation microcosms. Amended with Ca-citrate/Na-phosphate ((a) PPQ sediment (b) CR sediment), glycerol phosphate ((c) PPQ and (d) CR sediment) and polyphosphate ((e) PPQ sediment (f) CR sediment). Sorption control pH are also shown in (g) for PPQ sediment and CR sediments. Error bars are +/- one standard deviation, with microcosms run in triplicate.

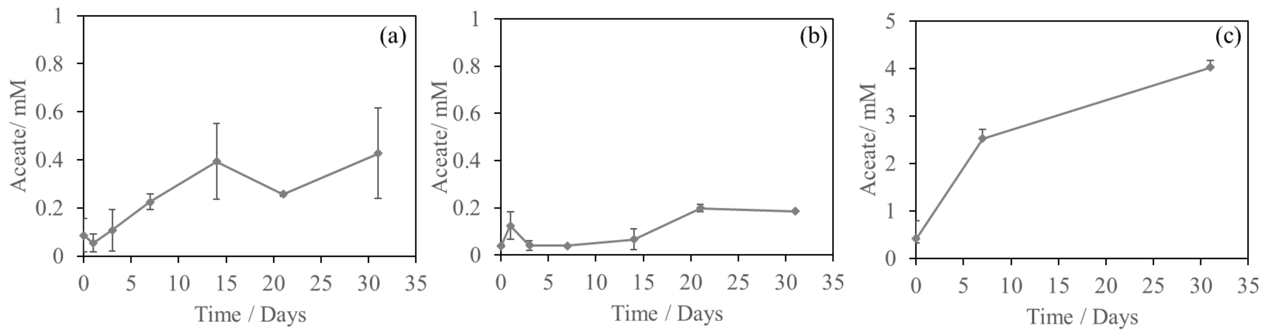

Figure S9. Ion chromatographic measurements of acetate from phosphate mineralisation microcosms. Amended with Ca-citrate/Na-phosphate; (a) PPQ sediment amended with 1 mM Ca^{2+}, 2.5 mM citrate and 10 mM phosphate, (b) 5 mM Ca^{2+}, 12.5 mM citrate and 10 mM phosphate. (c) CR sediment amended with 2 mM Ca^{2+}, 5 mM Citrate and 10 mM phosphate. Error bars are +/- one standard deviation, with microcosms run in triplicate.

Section S5: 16S rRNA Microbial Community Analysis

The raw data obtained in this research were deposited to NCBI SRA (Sequence Read Archive; http://www.ncbi.nlm.nih.gov/sra/) under the project accession number: PRJNA948651

PPQ Sediment Experiments

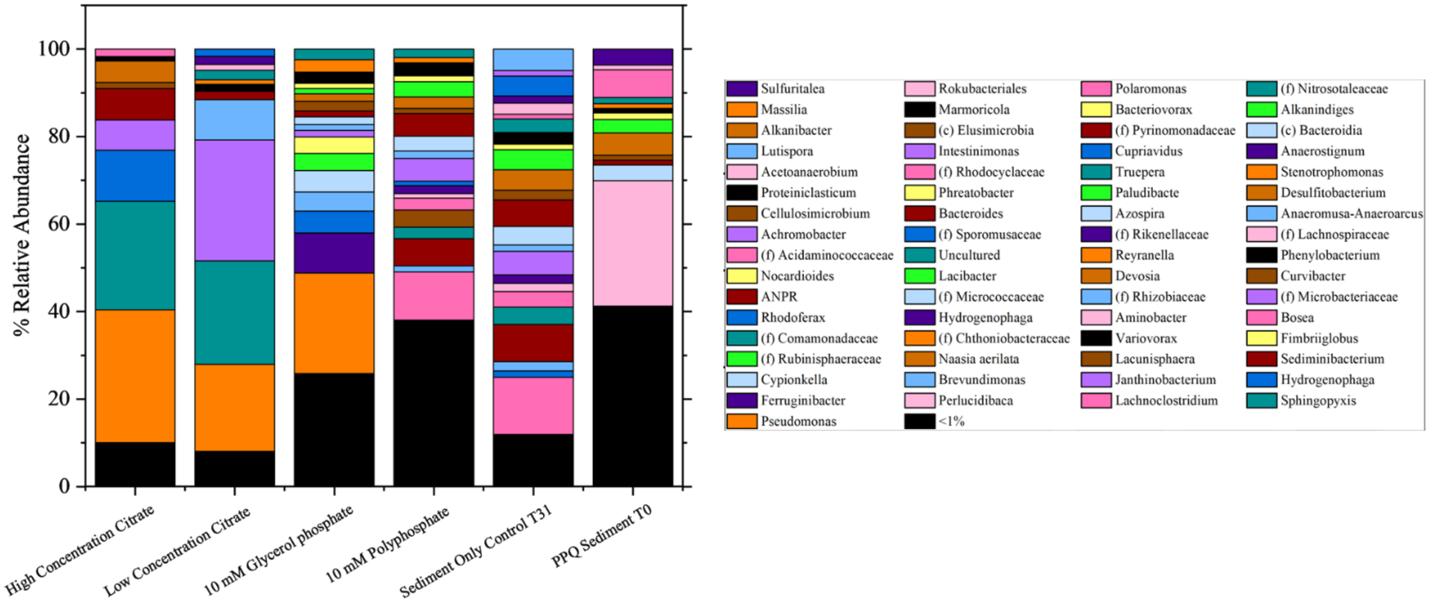

Figure S10. Data showing the microbial community structure at Genus level (>1% relative abundance) for PPQ sediment end points after 31 days of treatment with 5 mM Ca^{2+}, 12.5 mM Citrate and 10 mM phosphate (high concentration citrate), 1mM Ca^{2+}, 2.5 mM citrate and 10 mM phosphate (low concentration citrate), 10 mM glycerol phosphate, 10 mM polyphosphate and sediment only control. The initial starting peel place quarry (PPQ) sediment is also shown. Data is presented to the genus level, where the genus cannot be identified the family (f) or class (c) is given. ANPR (Allorhizobium-Neorhizobium-Pararhizobium-Rhizobium).

CR Sediment Experiments

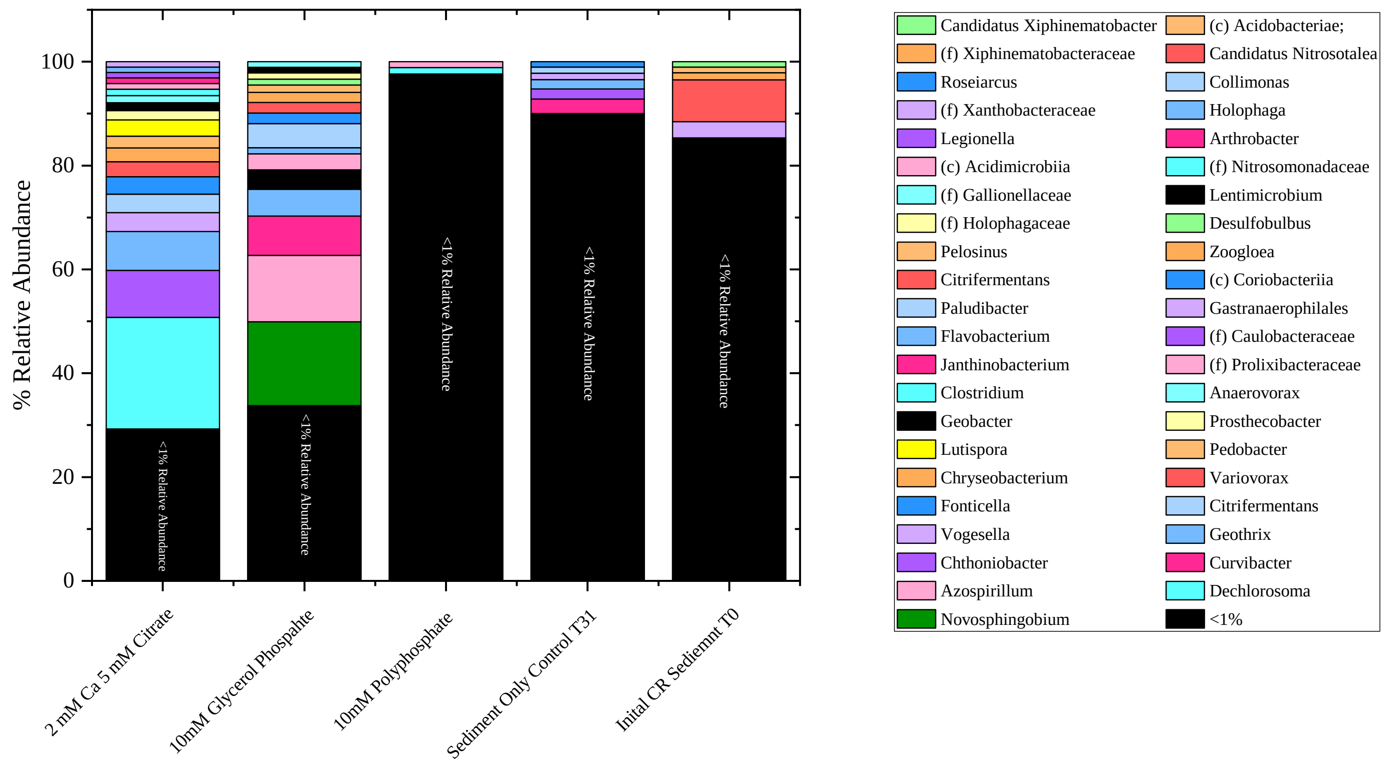

Figure S11. Data showing the microbial community structure at Genus level (>1% relative abundance) for CR sediment end points after 31 days of treatment with 2 mM Ca^{2+}, 5 mM Citrate and 10 mM phosphate, 10 mM glycerol phosphate, 10 mM polyphosphate and sediment only control. The initial starting Calder River (CR) sediment is also shown. Data presented to the genus level, where the genus cannot be identified the family (f) or class (c) is given.

Nucleotide Blast Results

Table S3. Results for 16S rRNA sequenced data from experimental systems, species were searched using the Basic Local Alignment Search Tool (BLAST) nucleotide search (http://blast.ncbi.nlm.nih.gov). A at >5% relative abundance. If species were unable to be identified information about the genus is given.

% Abundance in Sample	Name	Accession Number	% ID similarity	Score	Description	References
**Peel Place Quarry Sediment (PPQ)**						
**PPQ Citrate Amendment 5 mM Ca^{2+}, 12.5 mM citrate and 10 mM phosphate (high concentration citrate) Post Treatment (T_{31})**						
**24.8**	*g. Sphingopyxis*	NR_115617.1	100.0	440	Many species of aerobic and nitrate reducing soil bacteria from the genus *Sphingopyxi* were identified. This genus is commonly found in estuarine, marine and soil environments with numerus species (e.g *Sphingopyxis macrogoltabida*) capable of citrate assimilation.	[8,9]
**12.9**	*Pseudomonas (1) Sp. helmanticensis* strain OHA11	NR_126220.1	100.0	468	Sp *helmanticensis* strain OHA11 a strict aerobe isolated from forest soils a member of the genus *Pseudomonas,* which has been show to assimilate	[10]

					citrate and is a common soil bacteria.	
**11.7**	*Hydrogenophaga Sp. carboriunda* strain YZ2	NR_132726.1	99.6	462	Sp *carboriunda* facultative aerobe that can use citrate as an electron donor. Member of the genus *Hydrogenophaga*, which are common soli bacteria capable of carrying out heterotrophic nitrification and aerobic denitrification.	[11,12]
**9.9**	*Pseudomonas (2)* *Sp. Jessenii.* strain CIP 105274	NR_024918.1	100.0	468	Sp. *Jessenii* isolated form mineral waters and capable of citrate assimilation and nitrate reduction. Member of the genus *Pseudomonas* which are a common soli bacteria.	[13]
**7.6**	*Pseudomonas (3) Sp. prosekii* strain AN/28/1	NR_132724.1	100.0	468	*Sp prosekii* a citrate degrading species of the genus *pseudomonas*. Isolated form Antarctic rock/soil samples.	[14]
**7.2**	*Sediminibacterium goheungense*	NR_133854.1	100.0	468	Strict aerobic bacteria isolated	[15]

	strain HME7863				from fresh water reservoir.	
**6.9**	*Janthinobacterium rivuli* strain FT68W	NR_170540.1	100.0	468	An aerobe and facultative anaerobic spices of the genus *Janthinobacterium* isolated from a freshwater stream. This species is capable of metabolizing a wide range of organic molecules including glycerol, pyruvate and maltose.	[16]
**5.0**	*Naasia aerilata* strain 5116S-4 1	NR_109606.1	100.0	468	Air isolated bacteria capable of nitrate reduction and citrate assimilation.	[17]
**PPQ Citrate Amendment 1mM Ca2+, 2.5 mM citrate and 10 mM phosphate (low concentration citrate) Post Treatment (T_{31})**						
**27.6**	*Janthinobacterium rivuli* strain FT68W	NR_170540.1	100.0	468	An aerobe and facultative anaerobic spices of the genus *Janthinobacterium* isolated from a freshwater stream. Species as found in the high concentration citrate system.	[16]
**23.7**	*g. Sphingopyxis*	NR_115617.1	100.0	440	Many species of aerobic and nitrate reducing soil bacteria from the genus *Sphingopyxi* were identified as for the high concentration system. Many species of this	[8]

					genus can assimilate citrate under aerobic conditions.	
**10.3**	*Pseudomonas (2)* *Sp. Jessenii.* strain CIP 105274	NR_024918.1	100.0	468	Member of the genus *Pseudomonas* which are a common soli bacteria. Sp. *Jessenii* isolated form mineral waters, also found in the high concentration citrate system.	[13]
**9.3**	*Brevundimonas denitrificans* strain TAR-002	NR_133989.1	100.0	468	Aerobic denitrifying heterotrophic bacteria that can grow on citrate and was isolated from subsea sediments.	[18]
**7.2**	*Pseudomonas (1) Sp. helmanticensis* strain OHA11	NR_126220.1	100.0	468	Sp *helmanticensis* strain OHA11 a member of the genus *Pseudomonas,* which has been show to assimilate citrate and is a common soil bacteria.	[10]
**PPQ Glycerol Phosphate Amendment Post Treatment (T_{31})**						
**16.76**	*Pseudomonas (1) Sp. helmanticensis* strain OHA11	NR_126220.1	100.0	468	Sp *helmanticensis* strain OHA11 is a phosphate solubilising bacteria isolated from forests soils. From the genus *Pseudomonas* which can assimilate a variety of organic	[10]

					compounds including glycerol.	
**6.2**	*Pseudomonas (3) Sp. prosekii* strain AN/28/1	NR_132724.1	100.0	468	*Sp prosekii* a species of the genus *pseudomonas*. Isolated form Antarctic rock/soil samples.	14
**5.7**	*Ferruginibacter profundus* strain DS48-5-3	NR_148259.1	97.6	435	Aerobic bacteria with positive phosphatase activity. Isolated from freshwater river sediments.	19
**5.1**	*Hydrogenophaga Sp. carboriunda* strain YZ2	NR_132726.1	99.6	462	Sp *carboriunda* facultative aerobe. Member of the genus *Hydrogenophaga*, which are common soli bacteria capable of carrying out heterotrophic nitrification and aerobic denitrification.	11
**5.0**	*Cypionkella psychrotolerans* strain PAMC 27389	NR_148653.1	100.0	468	Facultative aerobic bacterial species isolated from terrestrial soils with positive phosphatase enzyme activity.	20
**PPQ Polyphosphate Amendment Post Treatment (T_{31})**						
**5.2**	*Achromobacter kerstersii strain LMG 3441*	NR_152015.1	100.0	468	A common soil bacteria.	21
**PPQ Sediment Only Control (T_{31})**						

**7.0**	*Sedimentibacter saalensis* strain ZF2	NR_025498.1	98.8	449	Dehalogenating bacterium capable for degrading tricholophenol. From the genus *Sedimentibacter* which are common soli bacteria.	[22]
**6.2**	*g. Lachnoclostridium*	NR_104899.1	100.0	468	Many species from the genus *Lachnoclostridium* were identified including *Hungatella xylanolytica* strain X5-1, which is closely related to *Clostridia*	(Yarza et al, 2013 Unpublished)
**5.4**	*Achromobacter kerstersii strain LMG 3441*	NR_152015.1	100.0	468	A common soil bacteria.	[21]
**Initial PPQ Sediment**						
**28.7**	*Perlucidibaca aquatica strain BK296*	NR_157660.1	100.0	468	A strict aerobe isolated from freshwater environment.	[23]
**Calder River Sediment (CR)**						
**CR Citrate Amendment Post Treatment (T_{31})**						
**21.5**	*Paraburkholderia denitrificans*	NR_108706.1	97.23	429	Facultative anaerobic bacteria capable of nitrate reduction isolated from forest soil samples. Other species of *Burkholderia*, such as *Burkholderia ferrariae* DSM 18251 can assimilate citrate.	[24]

**9.0**	*Chthoniobacter flavus Ellin428*	NR_115225.1	96.65	407	Bacteria isolated from soils of Rye grass which grows aerobically. The bacteria grows on many of the saccharides that can be found in plant biomass. It exclusively uses pyruvate as an organic carbon source, as it is unable to use other organic acids or amino acids.	[25]
**6.3**	*Geothrix fermentans strain H5 16S*	*NR_036779.1*	100.0	468	A species isolated from a contaminated aquifer. It can reduce Fe(III) through anaerobic respiration of organic acids and can ferment citrate. Can tolerate oxygen (<5%) but predominately and anaerobe. A member of the *acidobacteria* phylum, which contains a number of aerobic or facultative aerobic species, closely related to *Geothrix fermentans*	[26–28]
**CR Glycerol phosphate Amendment Post Treatment (T_{31})**						
**16.2**	*Novosphingobium humi strain R1-4*	NR_157799.1	100.0	468	Strictly aerobic bacteria, which possessive phosphatase enzyme activity	[29]

					and was isolated from soil.	
**12.8**	*Azospirillum thermophilum strain CFH 70021*	NR_170450.1	99.62	462	Aerobic bacteria capable of glycerol assimilation, it is phosphatase positive and was isolated from soil samples contaminated with hydrocarbons.	[30]
**7.6**	*Curvibacter lanceolatus strain NBRC 103051*	NR_114201.1	100.0	468	Bacteria capable of aerobic glycerol degradation. Isolated from a freshwater well.	[31,32]
**CR Polyphosphate Amendment Post Treatment (T_{31})**						
There were no species at >5% relative abundance in the polyphosphate treated system.						
**CR Sediment Only Control (T_{31})**						
There were no species at >5% relative abundance in the sediment only control system.						
**Initial CR Sediment (T_0)**						
There were no species at >5% relative abundance in the sediment only control system.						

Section S6: SEM/EDS Spot Images and EDS Elemental Mapping

Bulk PPQ Sediment SEM/EDS

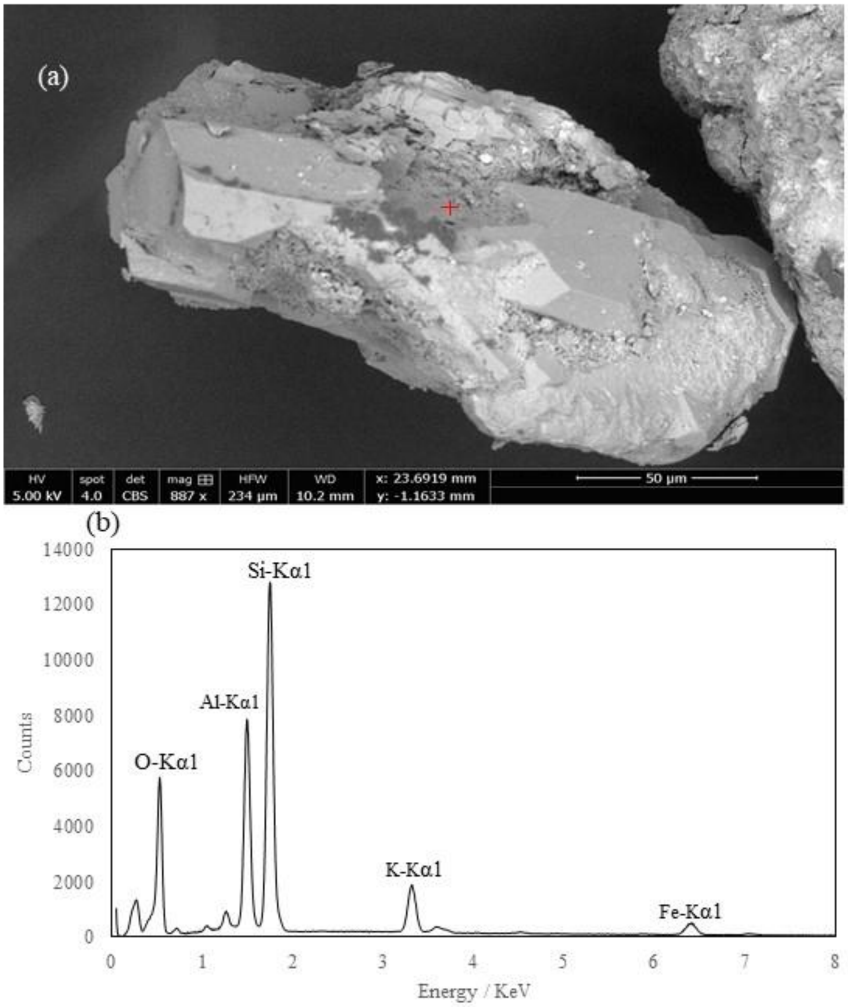

Figure S12. SEM image and associated spot EDS spectra from bulk PPQ sediment. With (a) showing iron oxide and feldspar coating a larger quartz grain and (b) is the associated spot EDS spectra.

Bulk CR Sediment SEM/EDS

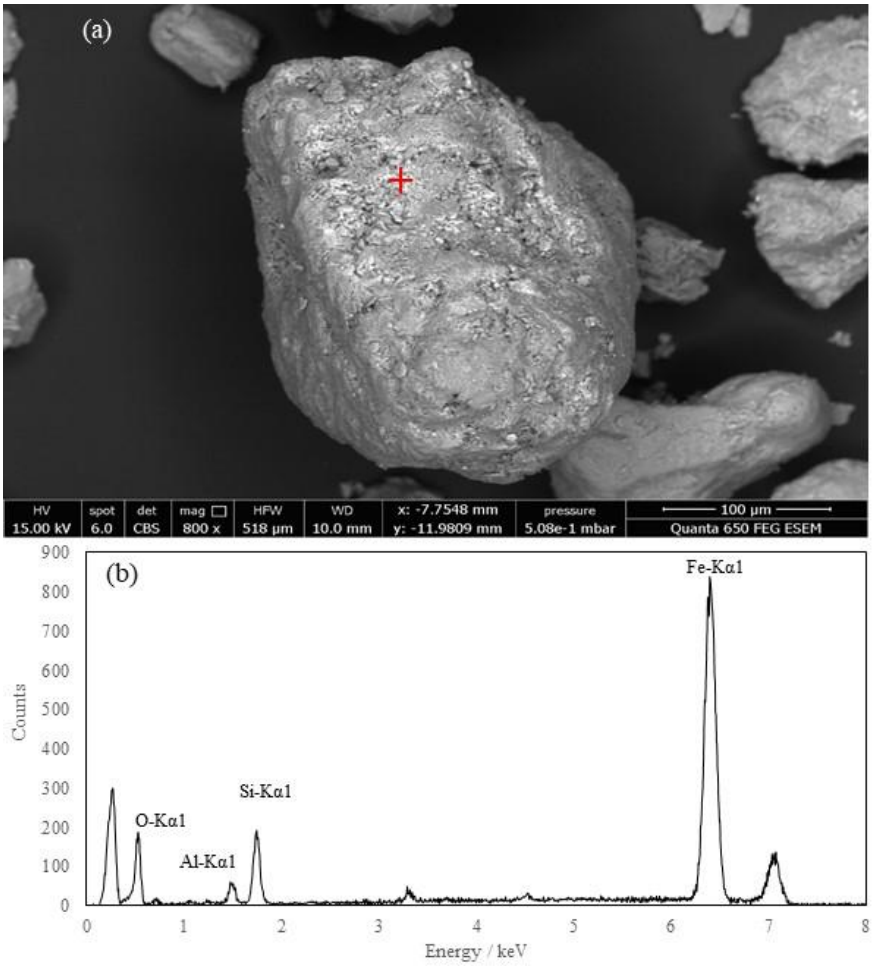

Figure S13. SEM image and associated spot EDS spectra from bulk CR sediment. With (a) showing iron rich despot coating a larger quartz grain and (b) is the associated spot EDS spectra.

EDS Mapping

Scanning electron microscopy and energy dispersive X-Ray spectroscopy was conducted on PPQ sediments from the low concentration Ca-citrate/Na-phosphate (1 mM Ca^{2+}, 2.5 mM citrate and 10 mM), 10 mM glycerol phosphate and 10 mM polyphosphate systems. For the CR microcosms only the Ca-citrate/Na-phosphate (2 mM Ca^{2+}, 5 mM citrate and 10 mM phosphate) amendment system was selected from SEM/EDS aalysis. Sediments were removed after 31 days, to investigate the morphology of precipitated biominerals and determine the speciation of Sr after treatment with amendment solutions. Additionally, EDS mapping was conducted to determine if Ca, P and Sr within the predicated biominerals were co-located.

PPQ Sediment.

Ca-citrate/Na-phosphate (1 mM Ca^{2+}, 2.5 mM citrate and 10 mM phosphate)

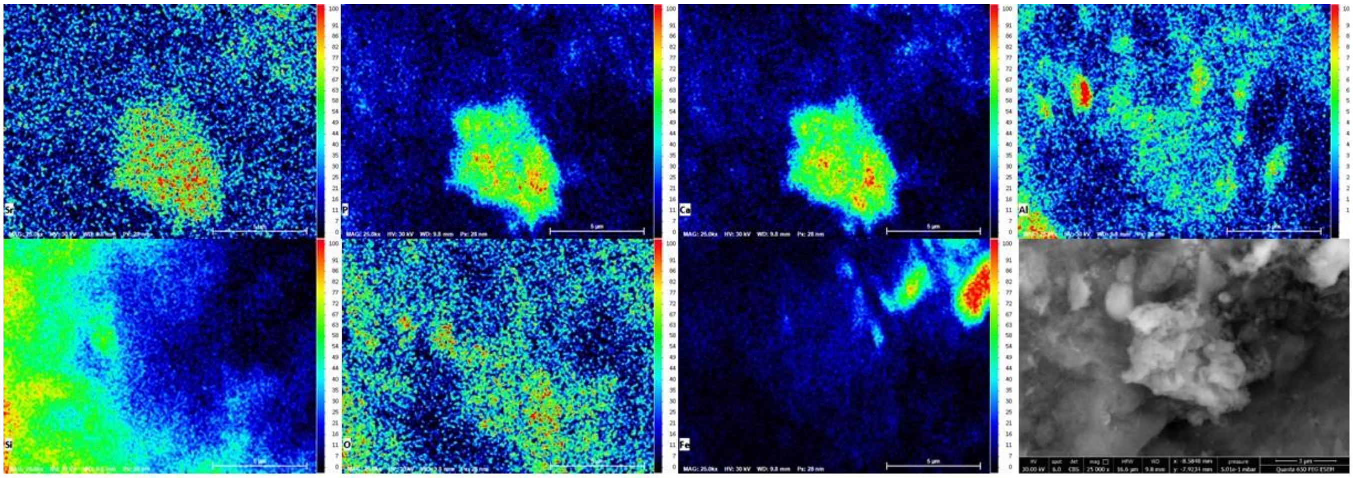

Figure S14. EDS mapping of PPQ sediment treated with Ca-citrate/Na-phosphate (1 mM Ca^{2+}, 2.5 mM citrate and 10 mM phosphate). Showing co-location of Ca, P and Sr within precipitated

10 mM Glycerol Phosphate.

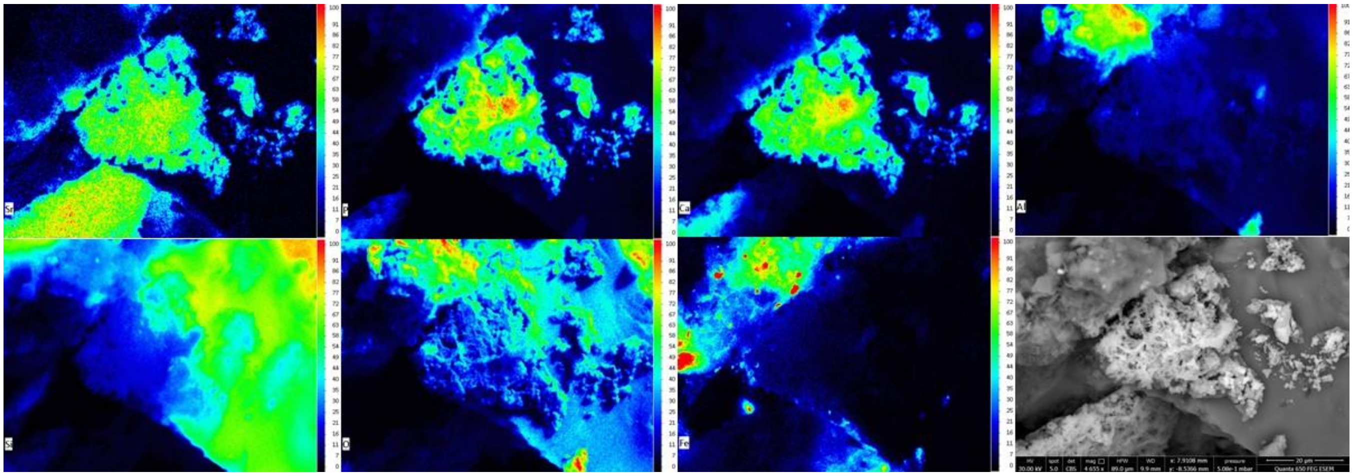

Figure S15. EDS mapping of PPQ sediment treated with 10 mM glycerol phosphate. Showing co-location of Ca, P and Sr within precipitated biominerals.

10 mM Polyphosphate.

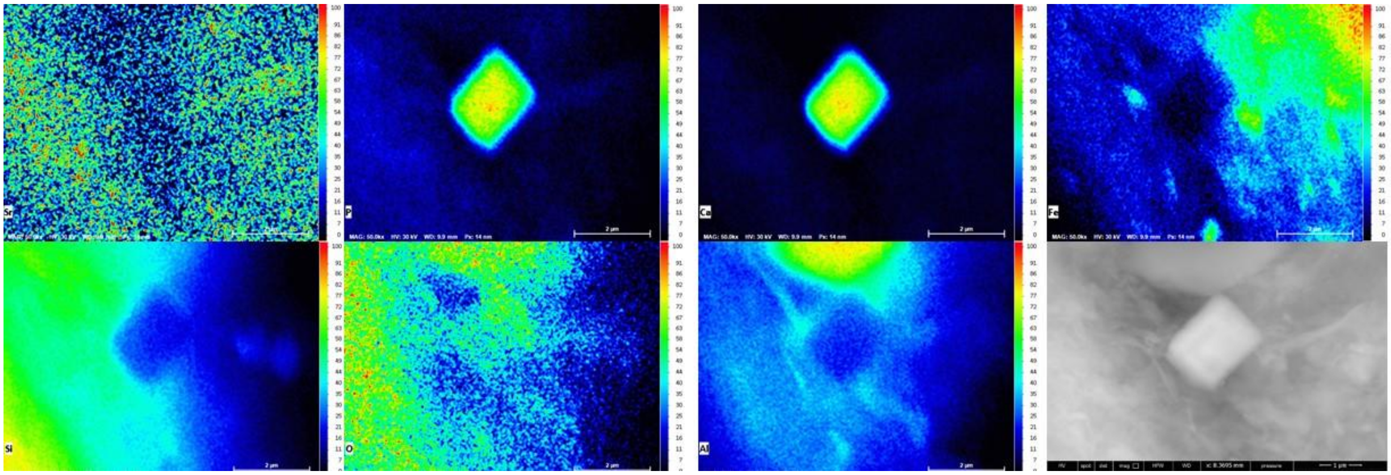

Figure S16. EDS mapping of PPQ sediment treated with 10 mM polyphosphate solution. Showing precipitated calcium phosphate minerals.

CR Sediment.

Ca-citrate/Na-phosphate (2 mM Ca^{2+}, 5 mM citrate and 10 mM phosphate).

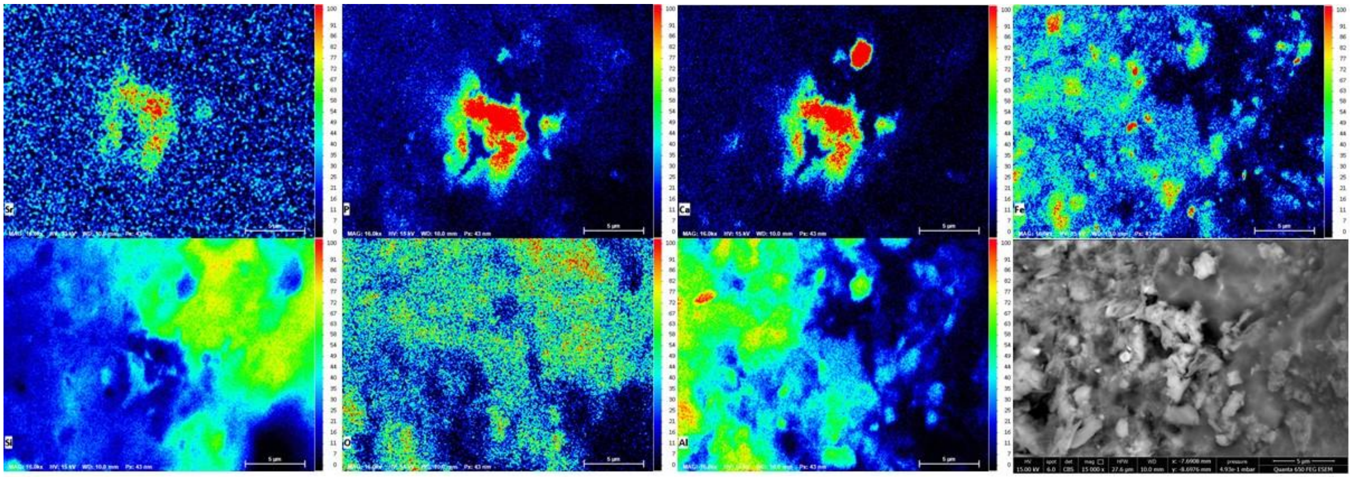

Figure S17. EDS mapping of CR sediment treated with Ca-citrate/Na-phosphate (2 mM Ca^{2+}, 5 mM citrate and 10 mM phosphate). Showing co-location of Ca, P and Sr within precipitated biominerals.

Section S7: XAS Fitting Parameters

XAS Fitting Parameters

N is shell occupancy, R(Å) is interatomic distance, σ^2 (Å^2) is the Debye-Waller factor, $S_O{}^2$ amplitude factor, R (least squared residual) goodness of fit factor, ΔE_0 denotes the energy shift (calculated) from the fermi level and α denotes the statistical significance of each shell from the F-test, determined from whether the fit was significantly worsened on removal of an individual shell.[33]

Table S3. Sr EXAFS Fitting parameters for the PPQ sediment microcosms (A) sorption control, (B)Ca-citrate/Na-phosphate (1 mM Ca^{2+}, 2.5 mM citrate and 10 mM phosphate) and (C) glycerol phosphate treatments. (D) 4% Sr doped hydroxyapatite standard, synthesized following the methods of [5,6].

Treatment	Scattering Path	N	R (Å)	σ^2 (Å^2)	$S_O{}^2$	R-factor	ΔE_0	F-Test (α) %
**Sorption Control (A)**	Sr-O	9.0	2.601 ± 0.012	0.012 ± 0.001	1	0.017	-3.4	-
**Ca-Citrate/Na-Phosphate (B)**	Sr-O	9.0	2.609 ± 0.011	0.012 ± 0.001	1	0.013	-0.38	100
	Sr-P	2.0	3.275 ± 0.017	0.009 ± 0.002	1			100
	Sr-Ca	0.9	4.104 ± 0.072	0.012 ± 0.011	1			66
**GlyPO4 (C)**	Sr-O	9.0	2.604 ± 0.010	0.011 ± 0.001	1	0.012	-1.6	100
	Sr-P	2.0	3.273 ± 0.018	0.003 ± 0.001	1			100
	Sr-Ca	1.0	4.099 ± 0.036	0.006 ± 0.005	1			94
	Sr-O	9.0	2.55 ± 0.020	0.015 ± 0.001	1	0.015	0.77	

4% Sr doped hydroxyapatite standard (D)	Sr-P	3.0	3.25 ± 0.013	0.008 ± 0.002	1			
	Sr-P	3.0	3.63 ± 0.005	0.011 ± 0.005	1			
	Sr-Ca	3.0	4.05 ± 0.030	0.015 ± 0.001	1			

REFERENCES

(1) Wilkins, M. J.; Livens, F. R.; Vaughan, D. J.; Beadle, I.; Lloyd, J. R. The Influence of Microbial Redox Cycling on Radionuclide Mobility in the Subsurface at a Low-Level Radioactive Waste Storage Site. *Geobiology* **2007**, *5* (3), 293–301.

(2) Sellafield Ltd. *Groundwater Monitoring at Sellafield: Annual Data Review 2016*; 2016.

(3) Parkhurst, D. L.; Appelo, C. A. J. *Description of Input and Examples for PHREEQC Version 3: A Computer Program for Speciation, Batch-Reaction, One-Dimensional Transport, and Inverse Geochemical Calculations*; Reston, VA, 2013.

(4) Law, G. T. W.; Geissler, A.; Boothman, C.; Burke, I. T.; Livens, F. R.; Lloyd, J. R.; Morris, K. Role of Nitrate in Conditioning Aquifer Sediments for Technetium Bioreduction. *Environ. Sci. Technol.* **2010**, *44* (1), 150–155.

(5) Afshar, A.; Ghorbani, M.; Ehsani, N.; Saeri, M. R.; Sorrell, C. C. Some important factors in the wet precipitation process of hydroxyapatite, Materials & Design, **2003**, 24 (3), 197-202.

(6) Catros, S.; Guillemot, F.; Lebraud, E.; Chanseau, C.; Perez, S.; Bareille, R.; Amédée, J.; Fricain, J. C. Physico-chemical and biological properties of a nano-hydroxyapatite powder synthesized at room temperature, IRBM, **2010**, 31(4), 226-233

(7) Giffaut, E.; Grivé, M.; Blanc, P.; Vieillard, P.; Colàs, E.; Gailhanou, H.; Gaboreau, S.;

Marty, N.; Madé, B.; Duro, L. Andra Thermodynamic Database for Performance Assessment: ThermoChimie. *Appl. Geochemistry* **2014**, *49*, 225–236.

(8) Sharma, M.; Khurana, H.; Singh, D. N.; Negi, R. K. The Genus Sphingopyxis: Systematics, Ecology, and Bioremediation Potential - A Review. *J. Environ. Manage.* **2021**, *280* (December 2020), 111744.

(9) Yun, N. R.; Shin, Y. K.; Hwang, S. Y.; Kuraishi, H.; Sugiyama, J.; Kawahara, K. Chemotaxonomic and Phylogenetic Analyses of Sphingomonas Strains Isolated from Ears of Plants in the Family Gramineae and a Proposal of Sphingomonas Roseoflava Sp. Nov. *J. Gen. Appl. Microbiol.* **2000**, *46* (1), 9–18.

(10) Ramírez-Bahena, M. H.; Cuesta, M. J.; Flores-Félix, J. D.; Mulas, R.; Rivas, R.; Castro-Pinto, J.; Brañas, J.; Mulas, D.; González-Andrés, F.; Velázquez, E.; Peix, Á. Pseudomonas Helmanticensis Sp. Nov., Isolated from Forest Soil. *Int. J. Syst. Evol. Microbiol.* **2014**, *64* (PART 7), 2338–2345.

(11) Reinauer, K. M.; Popovic, J.; Weber, C. D.; Millerick, K. A.; Kwon, M. J.; Wei, N.; Zhang, Y.; Finneran, K. T. Hydrogenophaga Carboriunda Sp. Nov., a Tertiary Butyl Alcohol-Oxidizing, Psychrotolerant Aerobe Derived from Granular-Activated Carbon (GAC). *Curr. Microbiol.* **2014**, *68* (4), 510–517.

(12) Yao, S.; Ni, J.; Ma, T.; Li, C. Heterotrophic Nitrification and Aerobic Denitrification at Low Temperature by a Newly Isolated Bacterium, Acinetobacter Sp. HA2. *Bioresour. Technol.* **2013**, *139*, 80–86.

(13) Verhille, S.; Baida, N.; Dabboussi, F.; Izard, D.; Leclerc, H. Taxonomic Study of Bacteria

Isolated from Natural Mineral Waters: Proposal of Pseudomonas Jessenii Sp. Nov. and Pseudomonas Mandelii Sp. Nov. *Syst. Appl. Microbiol.* **1999**, *22* (1), 45–58.

(14) Kosina, M.; Barták, M.; Mašlaňová, I.; Pascutti, A. V.; Šedo, O.; Lexa, M.; Sedláček, I. Pseudomonas Prosekii Sp. Nov., a Novel Psychrotrophic Bacterium from Antarctica. *Curr. Microbiol.* **2013**, *67* (6), 637–646.

(15) Kang, H.; Kim, H.; Lee, B. Il; Joung, Y.; Joh, K. Sediminibacterium Goheungense Sp. Nov., Isolated from a Freshwater Reservoir. *Int. J. Syst. Evol. Microbiol.* **2014**, *64* (PART 4), 1328–1333.

(16) Lu, H.; Deng, T.; Cai, Z.; Liu, F.; Yang, X.; Wang, Y.; Xu, M. Janthinobacterium Violaceinigrum Sp. Nov., Janthinobacterium Aquaticum Sp. Nov. and Janthinobacterium Rivuli Sp. Nov., Isolated from a Subtropical Stream in China. *Int. J. Syst. Evol. Microbiol.* **2020**, *70* (4), 2719–2725.

(17) Weon, H. Y.; Kim, S. J.; Jang, Y. H.; Hamada, M.; Tamura, T.; Ahn, J. H.; Suzuki, K. I.; Kwon, S. W. Naasia Aerilata Gen. Nov., Sp. Nov., a Member of the Family Microbacteriaceae Isolated from Air. *Int. J. Syst. Evol. Microbiol.* **2013**, *63* (PART7), 2436–2441.

(18) Tsubouchi, T.; Koyama, S.; Mori, K.; Shimane, Y.; Usui, K.; Tokuda, M.; Tame, A.; Uematsu, K.; Maruyama, T.; Hatada, Y. Brevundimonas Denitrificans Sp. Nov., a Denitrifying Bacterium Isolated from Deep Subseafloor Sediment. *Int. J. Syst. Evol. Microbiol.* **2014**, *64*, 3709–3716.

(19) Jin, L.; Lee, H. G.; La, H. J.; Ko, S. R.; Ahn, C. Y.; Oh, H. M. Ferruginibacter Profundus

Sp. Nov., a Novel Member of the Family Chitinophagaceae, Isolated from Freshwater Sediment of a Reservoir. *Antonie van Leeuwenhoek, Int. J. Gen. Mol. Microbiol.* **2014**, *106* (2), 319–323.

(20) Lee, Y. M.; Yang, J. Y.; Baek, K.; Han, S. J.; Shin, S. C.; Hwang, C. Y.; Hong, S. G.; Lee, H. K. Pseudorhodobacter Psychrotolerans Sp. Nov., a Psychrotolerant Bacterium Isolated from Terrestrial Soil, and Emended Description of the Genus Pseudorhodobacter. *Int. J. Syst. Evol. Microbiol.* **2016**, *66* (2), 1068–1073.

(21) Vandamme, P. A.; Peeters, C.; Inganäs, E.; Cnockaert, M.; Houf, K.; Spilker, T.; Moore, E. R. B.; LiPuma, J. J. Taxonomic Dissection of Achromobacter Denitrificans Coenye et Al. 2003 and Proposal of Achromobacter Agilis Sp. Nov., Nom. Rev., Achromobacter Pestifer Sp. Nov., Nom. Rev., Achromobacter Kerstersii Sp. Nov. and Achromobacter Deleyi Sp. Nov. *Int. J. Syst. Evol. Microbiol.* **2016**, *66* (9), 3708–3717.

(22) Breitenstein, A.; Saano, A.; Salkinoja-Salonen, M.; Andreesen, J. R.; Lechner, U. Analysis of a 2,4,6-Trichlorophenol-Dehalogenating Enrichment Culture and Isolation of the Dehalogenating Member Desulfitobacterium Frappieri Strain TCP-A. *Arch. Microbiol.* **2001**, *175* (2), 133–142.

(23) Baek, K.; Han, J. H.; Lee, M. H. Perlucidibaca Aquatica Sp. Nov., Isolated from Fresh Water. *Int. J. Syst. Evol. Microbiol.* **2017**, *67* (7), 2296–2300.

(24) Lee, C. M.; Weon, H. Y.; Yoon, S. H.; Kim, S. J.; Koo, B. S.; Kwon, S. W. Burkholderia Denitrificans Sp. Nov., Isolated from the Soil of Dokdo Island, Korea. *J. Microbiol.* **2012**, *50* (5), 855–859.

(25) Sangwan, P.; Chen, X.; Hugenholtz, P.; Janssen, P. H. Chthoniobacter Flavus Gen. Nov., Sp. Nov., the First Pure-Culture Representative of Subdivision Two, Spartobacteria Classis Nov., of the Phylum Verrucomicrobia. *Appl. Environ. Microbiol.* **2004**, *70* (10), 5875–5881.

(26) Lonergan, D. J.; Jenter, H. L.; Coates, J. D.; Phillips, E. J. P.; Schmidt, T. M.; Lovley, D. R. Phylogenetic Analysis of Dissimilatory Fe(III)-Reducing Bacteria. *J. Bacteriol.* **1996**, *178* (8), 2402–2408.

(27) Coates, J. D.; Ellis, D. J.; Gaw, C. V.; Lovley, D. R. Geothrix Fermentans Gen. Nov., Sp. Nov., a Novel Fe(III)-Reducing Bacterium from a Hydrocarbon-Contaminated Aquifer. *Int. J. Syst. Bacteriol.* **1999**, *49* (4), 1615–1622.

(28) Mehta, Misha Girish. Metal reduction by Geothrix fermentans. University of Minnesota, **2013**. Thesis retrieved from the University of Minnesota Digital Conservancy, https://hdl.handle.net/11299/1588632013.

(29) Hyeon, J. W.; Kim, K.; Son, A. R.; Choi, E.; Lee, S. K.; Jeon, C. O. Novosphingobium Humi Sp. Nov., Isolated from Soil of a Military Shooting Range. *Int. J. Syst. Evol. Microbiol.* **2017**, *67* (8), 3083–3088.

(30) Young, C. C.; Hupfer, H.; Siering, C.; Ho, M. J.; Arun, A. B.; Lai, W. A.; Rekha, P. D.; Shen, F. T.; Hung, M. H.; Chen, W. M.; Yassin, A. F. Azospirillum Rugosum Sp. Nov., Isolated from Oil-Contaminated Soil. *Int. J. Syst. Evol. Microbiol.* **2008**, *58* (4), 959–963.

(31) LEIFSON, E. The Bacterial Flora of Distilled and Stored Water. III. New Species of the Genera Corynebacterium, Flavobacterium, Spirillum and Pseudomonas. *Int. Bull. Bacteriol. Nomencl. Taxon.* **1962**, *12* (4), 161–170.

(32) Ding, L.; Yokota, A. Proposals of Curvibacter Gracilis Gen. Nov., Sp. Nov. and Herbaspirillum Putei Sp. Nov. for Bacterial Strains Isolated from Well Water and Reclassification of [Pseudomonas] Huttiensis, [Pseudomonas] Lanceolata, [Aquaspirillum] Delicatum and [Aquaspirillum. *Int. J. Syst. Evol. Microbiol.* **2004**, *54* (6), 2223–2230.

(33) Downward, L.; Booth, C. H.; Lukens, W. W.; Bridges, F. A Variation of the F-Test for Determining Statistical Relevance of Particular Parameters in EXAFS Fits. *AIP Conf. Proc.* **2007**, *882* (February 2007), 129–131.
